# Direct monitoring of single-cell response to biomaterials by Raman spectroscopy

**DOI:** 10.1007/s10856-021-06624-5

**Published:** 2021-12-04

**Authors:** Mary Josephine McIvor, Preetam K. Sharma, Catherine E. Birt, Hayley McDowell, Shannon Wilson, Stephen McKillop, Jonathan G. Acheson, Adrian R. Boyd, Brian J. Meenan

**Affiliations:** 1 Nanotechnology and Integrated Bioengineering Centre (NIBEC), School of Engineering, University of Ulster, Shore Road, Newtownabbey, Co. Antrim, BT37 0QB Northern Ireland UK; 2 Department of Chemical Engineering, Loughborough University, Loughborough, LE11 3TU England UK

## Abstract

There is continued focus on the development of new biomaterials and associated biological testing methods needed to reduce the time taken for their entry to clinical use. The application of Raman spectroscopy to the study of individual cells that have been in contact with biomaterials offers enhanced in vitro information in a potentially non-destructive testing regime. The work presented here reports the Raman spectral analysis of discreet U-2 OS bone cells after exposure to hydroxyapatite (HA) coated titanium (Ti) substrates in both the as-deposited and thermally annealed states. These data show that cells that were in contact with the bioactive HA surface for 7 days had spectral markers similar to those cultured on the Ti substrate control for the same period. However, the spectral features for those cells that were in contact with the annealed HA surface had indicators of significant differentiation at day 21 while cells on the as-deposited surface did not show these Raman changes until day 28. The cells adhered to pristine Ti control surface showed no spectral changes at any of the timepoints studied. The validity of these spectroscopic results has been confirmed using data from standard in vitro cell viability, adhesion, and proliferation assays over the same 28-day culture period. In this case, cell maturation was evidenced by the formation of natural bone apatite, which precipitated intracellularly for cells exposed to both types of HA-coated Ti at 21 and 28 days, respectively. The properties of the intracellular apatite were markedly different from that of the synthetic HA used to coat the Ti substrate with an average particle size of 230 nm, a crystalline-like shape and Ca/P ratio of 1.63 ± 0.5 as determined by SEM-EDX analysis. By comparison, the synthetic HA particles used as a control had an average size of 372 nm and were more-rounded in shape with a Ca/P ratio of 0.8 by XPS analysis and 1.28 by SEM-EDX analysis. This study shows that Raman spectroscopy can be employed to monitor single U-2 OS cell response to biomaterials that promote cell maturation towards de novo bone thereby offering a label-free in vitro testing method that allows for non-destructive analyses.

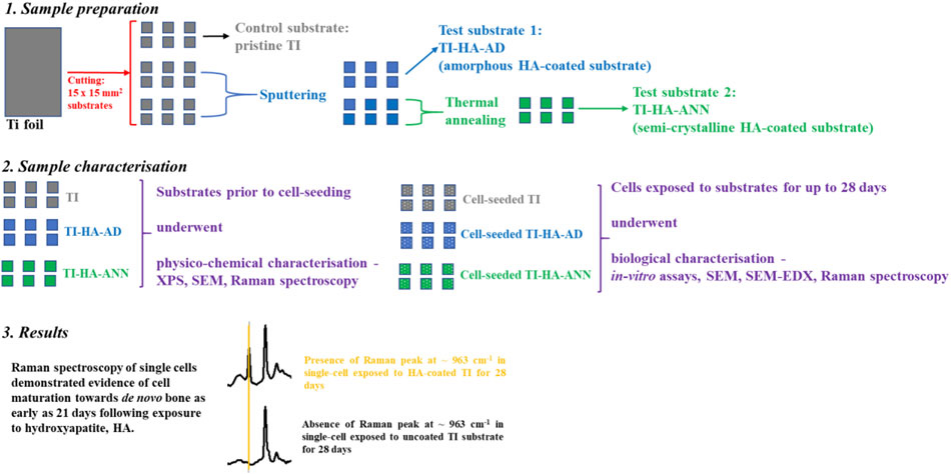

## Introduction

Methods to accurately test the biological response of cells to biomaterials, and the devices fabricated from them, are continually being developed. In the case of in vitro testing techniques, it is common to investigate the response of specific cells to a biomaterial in the context of a proposed clinical application for the attendant device such as, for tissue repair, restoration, or replacement purposes. In this context, the appropriate measure of biocompatibility must extend from bioinert to bioactive systems wherein cells adhere, proliferate and differentiate on the biomaterial or device depending on specific properties [1]. Hence, in vitro studies used to measure biocompatibility need to reflect the functional changes to cells, or lack thereof, that occur when they have been in contact with either active or inert biomaterials.

Titanium (Ti) is a common implantable metal widely used in the orthopaedic, cardiac, and dental fields. [2]. Commonly used medical grades of titanium are pure Ti (grade 2) and Ti6Al4V (grade 5) [3] both of which are deemed to be inherently bioinert. Notwithstanding its inherent properties, rigorous in vitro testing is required to confirm biocompatibility of Ti in the context of its application, as wear, corrosion and toxicity can occur post implantation. Surface modification and/or coating of Ti with other materials is commonly used to minimise or eliminate these issues. In this regard, hydroxyapatite (HA) (Ca_10_(PO_4_)_6_OH_2_) has been widely used as a coating on Ti to promote bone cell adhesion, proliferation and maturation and, ultimately to support the formation of functional bone tissue [4, 5]. This osteoconductive bioactive material is similar in chemical structure to bone apatite, it has a stoichiometric Ca/P ratio of 1.67 whereas, natural apatite is typically non-stoichiometric and either deficient in Ca or P, and it stimulates bone cells to attach, migrate and proliferate along its surface [6].

Typically, cell culture testing methods play a major role in fulfilling requirements of the standard in vitro cytotoxicity testing [7]. These methods fall into the main categories of immunochemical, biochemical, and molecular assays and, are typically time consuming and resource intensive. In addition, they are normally destructive in nature meaning that the cells being analysed cannot undergo on-going (sequential) in vitro testing or monitoring over a suitable time period. Such limitations can significantly reduce the speed at which new biomaterials reach commercial manufacture and clinical use. Hence, alternative complementary testing is needed for biomaterial biocompatibility testing that includes studies of cellular behaviour that better reflect the in vivo condition [8]. Raman spectroscopy has been shown to have attributes that can address some of these limitations of in vitro cell assays by offering a rapid, non-destructive, non-invasive and label-free analytical technique and has been used to monitor a variety of cell types, for both single and clustered cell analysis [9–12].

Raman spectroscopy operates on the basis of the Raman Effect, which occurs when light striking a material is scattered inelastically. The majority of light is scattered at the same wavelength as the incident source which is known as Rayleigh or elastic scattering. However, a small amount of scattered light (~0.0000001%) is scattered at different wavelengths depending on its interaction with the chemical bonds that are present in the material it interacts with and is referred to as Raman scattering. This provides a measurable output in the form of a Raman spectrum with intensity and wavelength position of the resulting peaks representing the vibration of a particular chemical bond. Raman spectroscopy employs lasers as the source of incident monochromatic light, which is typically coupled with a CCD camera to detect the scattered light and a microscope for high magnification visualisation of the sample and targeting of the location for spectral measurement. The Raman technique can also be combined with a confocal microscope optics to provide the benefits of variable depth of analysis.

In the work reported here, Confocal-Raman spectroscopy has been employed to study single U-2 OS bone cells cultured on pristine Ti and HA-coated Ti substrates in normal media. The spectral profile of cells has been studied after exposure to both as-deposited, sputter deposited, thin film hydroxyapatite (HA) coatings and after thermal annealing of the coated surface, with the differences between the two states confirmed by physico-chemical analyses. Standard in vitro cell assays have been performed to correlate cell phenotype with the Raman data in respect to (i) the effects of interaction with bioactive HA (ii) differences between the Raman spectra of U-2 OS cells cultured on as-deposited HA and annealed HA and (iii) the potential for Raman spectroscopy to assist in biocompatibility testing of biomaterials by correlation with data from standard biochemical assays.

## Material and methods

### Substrate preparation

Substrates were prepared from 1 mm thick Ti foil (99.7%, Sigma-Aldrich, Merck, UK). After cutting to the desired dimensions of 15 × 15 mm, they were abraded along each dimension using silicon carbide paper of decreasing grit size, then twice sonicated in propan-2-ol and once in deionised water. Substrates were dried using lint-free tissue followed by storage in a desiccator at room temperature where they remained until required. The abraded Ti substrates were placed into two groups; the first received no coating, referred to as uncoated Ti (TI) while the second set were coated with HA via Radio Frequency (RF) magnetron sputter deposition. This physical deposition coating process produces a homogenous HA-like calcium phosphate thin film on the upper surface of the Ti substrates. Sputter coating was performed in a custom-built high vacuum deposition system (Kurt. J. Lesker Ltd., UK), equipped with two Torus™ sputter sources at a 45° angle of incident angle to a sample holder located directly above. Each source is connected to a separate 13.56 MHz RF generator with an impedance matching network (Hüttinger, GmbH, Germany). Sputter targets of dimension 76 mm diameter and 5 mm thick were prepared from Captal^®^ ‘R’ Grade HA (Plasma Biotal Ltd., UK) using a manual hydraulic press (Specac, UK) to dry press ~12 g of powder at load of 40 kN for 10 min with loading rate of 10 kN per min to produce a target measuring. Targets were wrapped in aluminium foil and stored in a drying oven at 60 °C prior to being loaded into the Torus™ sputtering sources of the system.

To undertake sputter deposition, abraded Ti samples were placed in the sample holder and HA powder targets in the sources at a vertical distance of 100 mm. The vacuum chamber was allowed to reach a base pressure of 5 × 10^−5^ mbarr after which Argon gas (99.98%, BOC, UK) was introduced at a flow rate of 32–35 Sccm/min to achieve an operating pressure in the region 5 × 10^−2^ mbarr. The power to the two sources was ramped to 150 W at a rate of 1 W/s in order to avoid the targets cracking under thermal shock. Sputtering was then allowed to continue for a duration of 30 h after which the power was ramped down at 1 W/s and the system vented to atmospheric pressure under dry nitrogen. The HA-like coated substrates were placed in a desiccator at room temperature where they remained until required.

Coated substrates were placed into two groups of equal number; with the first set left in the as-deposited state (TI-HA-AD) while the second were placed on a high temperature ceramic tray and inserted into a furnace (AWF 12/5, Lenton 3216 furnace, Lenton, UK) to be annealed at 500 °C (at a heating rate of 10 °C per min) for 4 h [13, 14]. Annealing of the as-deposited coatings on the Ti substrates (TI-HA-AD) resulted in coating re-crystallisation (TI-HA-ANN). The sputtering and thermal annealing techniques described are well-established and proven in-house methods for preparing homogenous as-deposited HA-like thin film coatings and re-crystalised HA-like thin film coatings, respectively, on Ti and other substrates [14–17].

Prior to use in the in vitro cell assays, the required numbers of each substrate type (as-deposited and annealed) were individually wrapped in triple-layered aluminium foil, securely sealed with autoclave tape and placed in a dry oven (OV-12. Thermo Fisher Scientific, USA) at 160 °C for 4 h to remove any microbial load. The sterilised substrates were placed in a Class 2 Biosafety Cabinet (Bioquell UK Ltd., UK) under aseptic conditions and transferred to sterile 12-well tissue culture plastic plates until required. Quartz substrates (15 × 15 mm, Crystran Ltd., UK) required for the Raman analysis of single cells were also sterilised at the same time and transferred to culture plates as described above. All subsequent cell culture and associate biological assays were carried out under aseptic conditions.

### X-ray photoelectron spectroscopy (XPS)

X-ray photoelectron spectroscopy (XPS) is an analytical technique that provides information on chemical elements present within the upper surface (<10 nm) of a material. In this study, prior to cell-seeding, XPS was used to confirm the presence of HA coatings on the Ti substrates before and after thermal annealing and to give an indication of its distribution across the surface. The XPS system used a Kratos Axis Ultra with Delay Line Detector spectrometer (Kratos Analytical Ltd., UK). X-rays were generated at 15 kV voltage and 10 mA emission current. Monochromated Al Kα X-rays (hѵ = 1486.6 eV) were used to generate photoelectrons. A charge neutraliser system was employed operating at a filament current of 1.95 A and charge balance of 3.3 V.

Wide energy survey scans (WESS) were obtained from three random locations on each HA-coated substrate (Section “Substrate preparation”) at 160 eV pass energy followed by high-resolution spectra at 40 eV pass energy. Subsequent high-resolution spectra were then recorded for the titanium (Ti 2p), oxygen (O 1s), calcium (Ca 2p) carbon (C 1s) and phosphorus (P 2p) regions. Uncorrected charging effects on the calculated binding energy (BE) positions were adjusted by setting the lowest BE component of C 1s spectral envelope to 284.8 eV, a widely accepted value for adventitious carbon surface contamination on non-polymeric samples [18]. Peak fitting of the spectra was undertaken using CasaXPS Software (version 2.3.19PR1.0) (Casa Software Ltd., England, UK) by subtracting a Shirley background correction and applying a mixed Gaussian–Lorentzian synthetic peak function. Atomic concentration quantification was determined in the same software suite on the background corrected plots.

### Scanning electron microscopy (SEM)

Field emission scanning electron microscopy, FESEM, was undertaken in a Hitachi SU5000 instrument (Hitachi, UK) to study the surface topography of the Ti substrates before and after HA coating, and 28 days after cell-seeding (cell-seeding is described in Section “Cell-seeding”). The cell-seeded substrates at day 28 were preserved as follows; medium was removed from wells containing test substrates, wells washed twice with 0.01 M Phosphate Buffered Saline (PBS) and chemically fixed with 2.5% glutaraldehyde (Sigma-Aldrich, 230 Merck, UK) in water for 45 min at room temperature followed by two washes with 0.01 M PBS. Wells were gradually dehydrated using an alcohol series of increasing ethanol concentration, 25, 50, 75, 90% ethanol, for 8 min each at room temperature, followed by 100% ethanol for 8 min, twice. Wells were chemically dried overnight at room temperature with 100% hexamethyldisilizane.

Given the insulating nature of the HA-coated surfaces, an ultra-thin conductive gold layer was deposited onto all samples using an Emitech K500X coating system (Quorum Technologies, UK) operating at 25 mA for 150 s. SEM images were then acquired at an acceleration voltage of 10 kV for the Ti substrates before and after HA coating and, 5 kV after cell-seeding, a working distance of 6.7 mm and a nominal spot size of 100 nm at magnification settings ranging from x250 to x3000 times. Energy-dispersive X-ray (EDX) analysis was undertaken using a X-Max silicon drift detector (Oxford Instruments, UK) attached directly to the SEM analysis chamber with EDX analysis undertaken under the same operational conditions as the imaging with a setting of 5 frames/map recorded at a resolution of 1024 × 1024.

FESEM analysis was also carried out on substrates exposed to U-2 OS cells for 28 days as per the method described in Section “Single-cell analyses by Confocal-Raman spectroscopy and SEM with energy-dispersive X-ray (SEM-EDX)”.

### Raman spectroscopy

Raman spectroscopy was used to analyse the Ti substrates (i) before and after HA coating and (ii) 28 days after exposure to medium. In order to determine the direct effects of exposure to medium on the HA coatings, 2 ml of medium was added per well per substrate type within 12-well plates and incubated under standard conditions for 28 days with medium replenished every 3 days. Raman spectroscopy was used to analyse three random locations on three samples of each substrate type after exposure to medium under standard conditions for 28 days and compared with the spectra taken at day 0 to determine if the HA coating was still present. Each substrate type was analysed in duplicate. Prior to commencing measurements, the Raman system, a Renishaw inVia™ Qontor^®^ Confocal Raman Microscope (Renishaw Ltd., UK), was calibrated using an internal silicon reference to 520 cm^−1^. In acquisition mode, the laser was operated at 50% power (equal to 25 mW) and focused through a 50× long working distance (2 mm) objective over an extended wavenumber scan, 200–2000 cm^−1^, with 10 s integration time. Three spectra were recorded for each sample and subjected to a number of data processing steps which included; (i) cosmic ray removal if applicable, (ii) baseline subtraction, (iii) normalisation and (iv) smoothing, to remove any spectral artefacts and to render spectra comparable and ready for any further data analysis and interpretation. Data were normalised by intensity range using ‘1’ as the upper range and ‘0’ as the lower range. Spectral data were subsequently smoothed using Savitsky–Golay parameters; smooth window ‘5’ and polynomial order ‘2’. Raman data were plotted and processed using Origin Data Analysis and Graphical Software (OriginLab Corporation, USA).

### Cell culture

In this study, human osteosarcoma cells from a well-established immortalised cell-line, U-2 OS (HTB-96, ATCC, USA), were used as a ‘model’ for human bone cells. U-2 OSs were chosen over other commonly used osteosarcoma cells such as, MG-63s and Saos-2s, because in this study cell response over several weeks was investigated and the authors wanted to ensure the cells had the ability to demonstrate maturation characteristics such that they were able to mineralise to form bone-like nodules in vitro, which the authors have previously reported on with U-2 OSs using microscopy techniques and Raman spectroscopy [19]. Cells were cultured in McCoy’s 5A medium with L-glutamine (Sigma-Aldrich, Merck, UK) and supplemented with 10% (v/v) foetal bovine serum (Sigma-Aldrich, Merck, UK) and 1% (v/v) antibiotic mix (5000 units penicillin and 5 mg streptomycin/ml) (Sigma-Aldrich, Merck, UK), henceforth referred to as ‘medium’ and placed in a humidified incubator at 37 °C with 5% CO_2_ (standard conditions). Cells were maintained at below 70% confluency and passaged every 3 days using 0.05% trypsin with 0.02% ethylenediamine tetraacetic acid (EDTA) (Sigma-Aldrich, Merck, UK). Where possible cells were monitored using an inverted microscope (Nikon Eclipse TS100, Nikon, The Netherlands).

### Cell-seeding

At day 0, cells at passage number 11 had their concentration determined using an automated cell counter (TC20, Bio-Rad Laboratories Ltd., UK) as per the manufacturer’s protocol. Cell suspensions were standardised to 2 × 10^5^ cells per ml and 75 µl of this suspension (15,000 cells per substrate/10,000 cells per cm^2^ of substrate) pipetted onto the centre of each substrate within sterile 12-well plates. Cell-seeded substrates were incubated for 2 h under standard conditions to maximise cell adhesion, after which, 1925 µl of medium was added. Plates were incubated under standard conditions until each experimental timepoint was reached with medium replenished every 3 days, as necessary. Tissue culture plastic controls were included and albeit of different composition were treated in the same manner as that of the test substrates described above. Negative controls consisted of 75 µl of medium only (medium, no cells or substrate) and positive controls of 75 µl of cell suspension only (cells and medium, no substrate) were also employed. The negative controls were used to demonstrate maintenance of aseptic conditions, while the positive controls were used to demonstrate the merit of the cell line.

### In vitro cell assay for measuring cell metabolic activity

At days 1, 3, and 7 the metabolic activity of cells adhered on each substrate was quantified using 3-(4, 5-dimethylthiazol-2-yl)-2, 5-diphenyltetrazolium bromide) MTT colorimetric assay as a measure of cell viability, adhesion and proliferation. MTT solution, 5 mg/ml in 0.01 M PBS, was added to the wells containing test substrates and controls at 10% of the total well volume and incubated for 3 h at 37 °C in the dark. Wells were treated with 10% sodium dodecylsulfate (Sigma-Aldrich, Merck, UK) with 0.01 M hydrochloric acid (HCl) (Sigma-Aldrich, Merck, UK) and incubated overnight at room temperature. Wells were thoroughly mixed and aliquots (3 × 100 µl per well) transferred to a clear 96-well microplate from where their absorbance at a wavelength of 562 nm was measured using a Tecan Sunrise Spectrophotometer (Tecan Group Ltd., Switzerland). Each substrate type was tested in triplicate.

### In vitro cell assay for measuring deoxyribonucleic acid (DNA)

At days 1, 3, and 7 the DNA concentration of cells adhered on each substrate type was quantified using a commercial kit, Quant-iT™ Pico Green™ dsDNA (PG) Assay Kit (Thermo Fisher Scientific, UK). Medium was removed from wells containing test substrates and controls. Wells were then washed twice with 0.01 M PBS and any adhered cells released by adding a cell dissociation solution, Tryple Express (Thermo Fisher Scientific, UK) with 1% Triton X-100 (Sigma-Aldrich, Merck, UK) and incubating under standard conditions for 30 min. Cell recovery was maximised by using a pipettor to gently move the suspension up and down over the substrate at least five times. Cell suspension was transferred to a sterile microtube which was then frozen and thawed twice to maximise cell lysis and release of DNA into the suspension.

A DNA standard curve was prepared following the manufacturer’s protocol. Five standards, 1000, 100, 10, 1, and 0 ng/ml, were prepared using supplied kit reagents. PG fluorescent probe was prepared using supplied kit reagents and added to DNA (within standards and cell suspension samples) in a 1:1 ratio mixture from where they were thoroughly mixed and incubated for 5 min at room temperature in the dark. Following thorough mixing, aliquots of the solution (3 × 200 µl) were transferred to a black 96-well microplate and fluorescence measured using a Tecan Genios Spectrophotometer (Tecan Group Ltd., Switzerland) at an excitation wavelength of 480 nm and an emission wavelength of 540 nm. Each substrate type was tested in triplicate. Fluorescence intensity (arbitrary units) for the standards was plotted against DNA concentration (ng/ml), equation of the curve obtained (Microsoft Excel^®^, Microsoft Inc., USA) and the resulting standard curve used to calculate average DNA concentration per substrate type.

### Single-cell analyses by Confocal-Raman spectroscopy and SEM with energy-dispersive X-ray (SEM-EDX)

At days 7, 14, 21, and 28 medium was removed from wells containing test substrates and controls, wells washed twice with 0.01 M PBS and any adhered cells released by adding 500 µl of a cell dissociation solution, 0.05% trypsin with 0.02% EDTA, and incubating under standard conditions for 4–5 min. Cell recovery was maximised by gently pipetting the suspension up and down over the substrate at least five times. Cell suspension was transferred to sterile quartz substrates and 1500 µl of medium added to each well followed by overnight incubation under standard conditions. Wells were washed twice with 0.01 M PBS and chemically fixed using 100% methanol for 10 min at room temperature in preparation for Raman analysis. In this study, Confocal-Raman spectroscopy was used to analyse three random single cells on each cell-seeded quartz substrate at two cell locations, centre of the cell (assumed to be the nucleus) and outer region within the cell (assumed to be the cytoplasm) to confirm the presence of cells and to determine any significant spectral differences due to the study’s variables; (i) use of three types of Ti substrate, (ii) culture time and (iii) cell location. A laser beam at 10% power (equal to 5 mW) was focused through a 50× long working distance (2 mm) objective over an extended wavenumber scan, 600–1800 cm^−1^, with 120 s integration time per spectrum. Twelve replicates were performed for each spectrum. Spectra were subjected to the same data processing steps as described in Section “Raman spectroscopy” along with subtraction of the Raman spectrum owing to the quartz substrate.

Quartz samples at day 21 and 28 were then further characterised using SEM-EDX to obtain elemental composition of single cells derived from TI, TI-HA-AD, and TI-HA-ANN at day 21 and 28 at an acceleration voltage of 10 kV. Elements were selected from considering the sample under test (i) quartz (containing silicon (Si) and O) and (ii) cells (primarily containing C, nitrogen (N), P, and O). In addition, sodium (Na) and chlorine (Cl) were selected due to the high use of PBS in biological methods and Ca as an indicator of bone mineralisation. SEM-EDX was operated under low vacuum and electron backscattered diffraction mode due to the non-conductive nature of quartz samples and the desire to avoid gold-palladium-sputtering to allow for repeat Raman analyses if required. Where possible, a minimum of three single cells per quartz sample were analysed and their element composition (as atomic concentration, at %) attained using Aztec Nanoanalysis Software (Oxford Instruments Plc., UK).

### Statistical analyses

Statistical analyses for the MTT and PG assay results (*n* = 3 minimum) were performed using GraphPad Prism version 8 for Windows (GraphPad Software, USA). Any significant difference in the results for cells seeded and cultured on the uncoated substrate (TI) to those on the two types of HA-coated Ti substrates (TI-HA-AD and TI-HA-ANN) was determined using Dunnett’s multiple comparison test with a value of *P* < 0.05 taken as statistically significant.

## Results

### HA coating

**Fig. 1:**
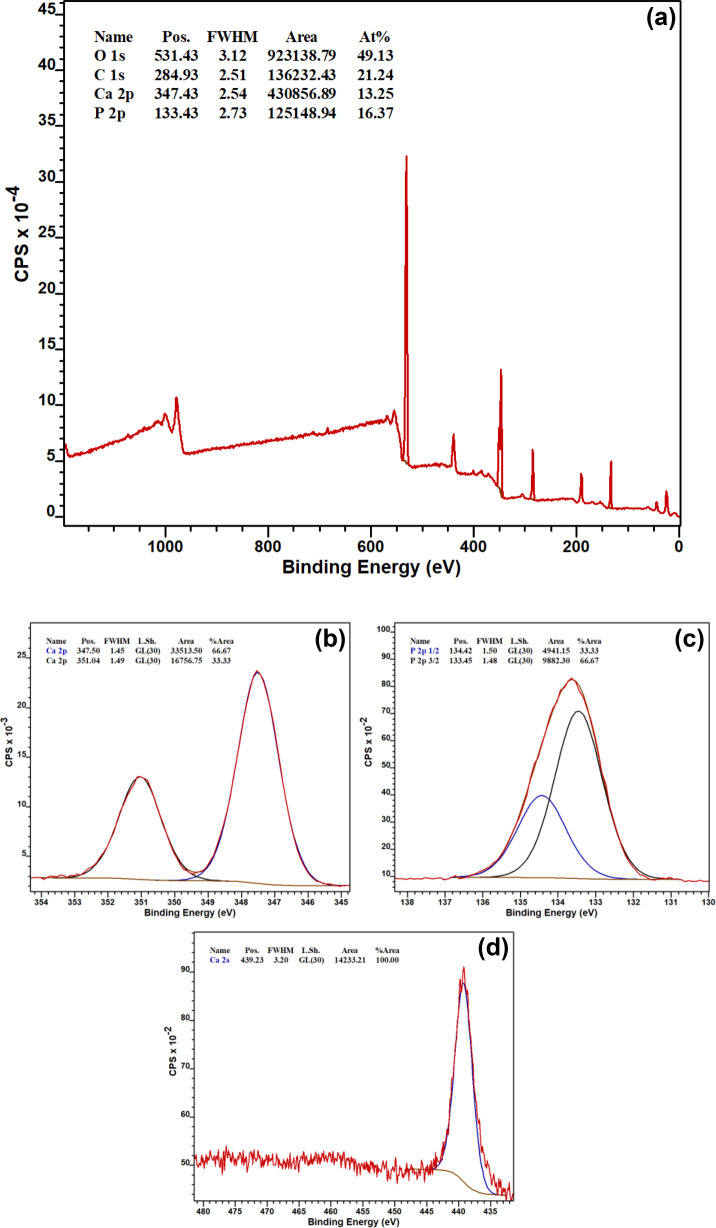
**a** XPS WESS from one random location on HA-coated Ti substrate. XPS high resolution scans for elemental orbitals **b** Ca 2p, **c** P 2p, and **d** Ti 2p from one random location on HA-coated Ti substrate

Physico-chemical characterisation of Ti substrates at day 0, prior to cell-seeding, by XPS, SEM, and Raman spectroscopy was used to confirm the HA coating on Ti substrates (Fig. 1 and Supplementary Information Figs. 1, 2, respectively).

The presence of XPS peaks namely Ca 2p, O 1s and P 2p confirmed that CaP, in this case HA, was successfully coated onto Ti substrates with Ca 2p and P 2p peaks at 347.43 and 133.43 eV with atomic concentrations of 13.25% and 16.37%, respectively (Fig. 1a). From atomic concentrations, Ca/P ratio was 0.8 and peaks representative of Ti (BE ~460 eV) were not obtained suggesting a homogeneous coating across the upper surface (Fig. 1a–c). P at 439 eV corresponds to Ca 2s (Fig. 1d).

The SEM image for uncoated substrate (TI) has prominent features of unidirectional linear striations which are in stark contrast to that of HA-coated Ti substrate where a change in surface topography from the addition of coating is evident; unidirectional linear striations are no longer visible (Supplementary Information Fig. 1a, b, respectively). Supplementary Information Fig. 1b shows uniform coverage of relatively round and clustered particles giving the surface what appears to be a more uneven topography/roughness resulting in randomly orientated nano-sized pores and pits. Supplementary Information Fig. 1c is another representation of HA coating, with average particle size 372 nm (particles size range 280–520 nm) (Supplementary Information Fig. 1e). EDX maps were collected from the same area as Supplementary Information Fig. 1c and show peaks from different elements, namely O, P, and Ca (Supplementary Information Fig. 1d) with a Ca/P ratio of 1.28. Contrary to the XPS result stated above, Ti was detected (Supplementary Information Fig. 1d).

Further evidence of successful coating of HA was obtained by performing Raman spectroscopy on three random locations on each substrate type, TI, TI-HA-AD, and TI-HA-ANN (Supplementary Information Fig. 2a–c). Three replicates were performed for each spectrum taken. Raman peak at ~960 cm^−1^ was present for both TI-HA-AD and TI-HA-ANN (Supplementary Information Fig. 2b, c, respectively) which is indicative of the P–O symmetric stretch (v_1_) within the phosphate group, PO_4_^3−^ present within HA [20]. As expected, no such peak was present in TI (Supplementary Information Fig. 2a).

In addition, Raman spectroscopy was used to chemically characterise three substrate types after exposure to medium under standard conditions for 28 days (Supplementary Information Fig. 3a–c). Raman peak at ~960 cm^−1^ was present for both TI-HA-AD and TI-HA-ANN (Supplementary Information Fig. 3b, c, respectively) with similar intensities to those at day 0, indicating that the HA coating remained on both the as-deposited and thermally annealed substrates after 28 days in medium. As expected, no such peak was present in TI (Supplementary Information Fig. 3a).

### SEM

**Fig. 2:**
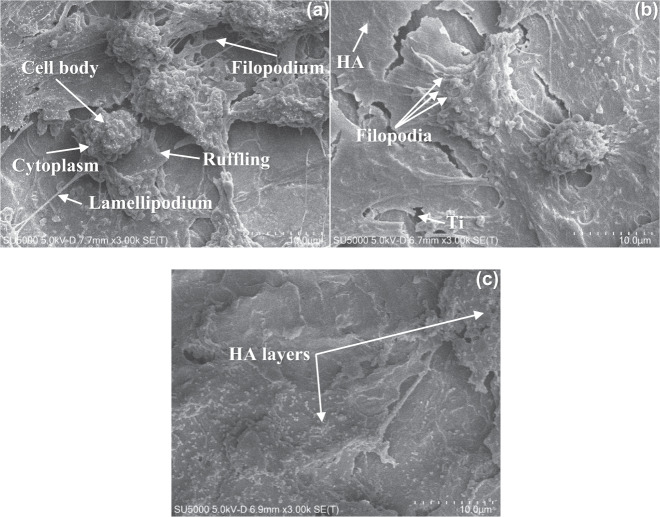
Annotated SEM images taken at day 28 from one random location on **a** TI, **b** TI-HA-AD and **c** TI-HA-ANN at 3 k magnification. Scale bar represents 10 µm distance within image

A visual perspective of SEM images of the three substrate types taken at day 28 at 3 k magnification gives qualitative information on their biological potential to support cells (Fig. 2). Cells are visible on TI (Fig. 2a) and TI-HA-AD (Fig. 2b) but not on TI-HA-ANN (Fig. 2c). Numerous attempts were made to locate cells on TI-HA-ANN. While cell morphology is broadly similar between cells shown for TI and TI-HA-AD, cells on TI have more pronounced cell bodies while those on TI-HA-AD have flatter, more elongated appearance. For both images, the main cell features expected at this magnification are visible; cell body, cytoplasm, ruffling, filopodia and lamellipodia. Filopodia are observed interrogating the substrate surface for anchorage and show more cells on TI than on TI-HA-AD (Fig. 2a, b, respectively).

As described in Section “HA coating”, HA remained on HA-coated substrates, TI-HA-AD and TI-HA-ANN, after 28 days in culture. SEM images for TI-HA-AD (Fig. 2b) and TI-HA-ANN (Fig. 2c) provide further evidence of this result; HA is visible, images show a ‘break’ in HA coating exposing the underlying Ti substrate. A ‘flake-like’ appearance of HA is visible for TI-HA-AD (Fig. 2b). However, the uniform coverage of relatively round and clustered particles observed at the same magnification in Supplementary Information Fig. 1b is no longer present; a flatter, more ‘flake-like’ and layered appearance is observed.

### In vitro cell assays

**Fig. 3:**
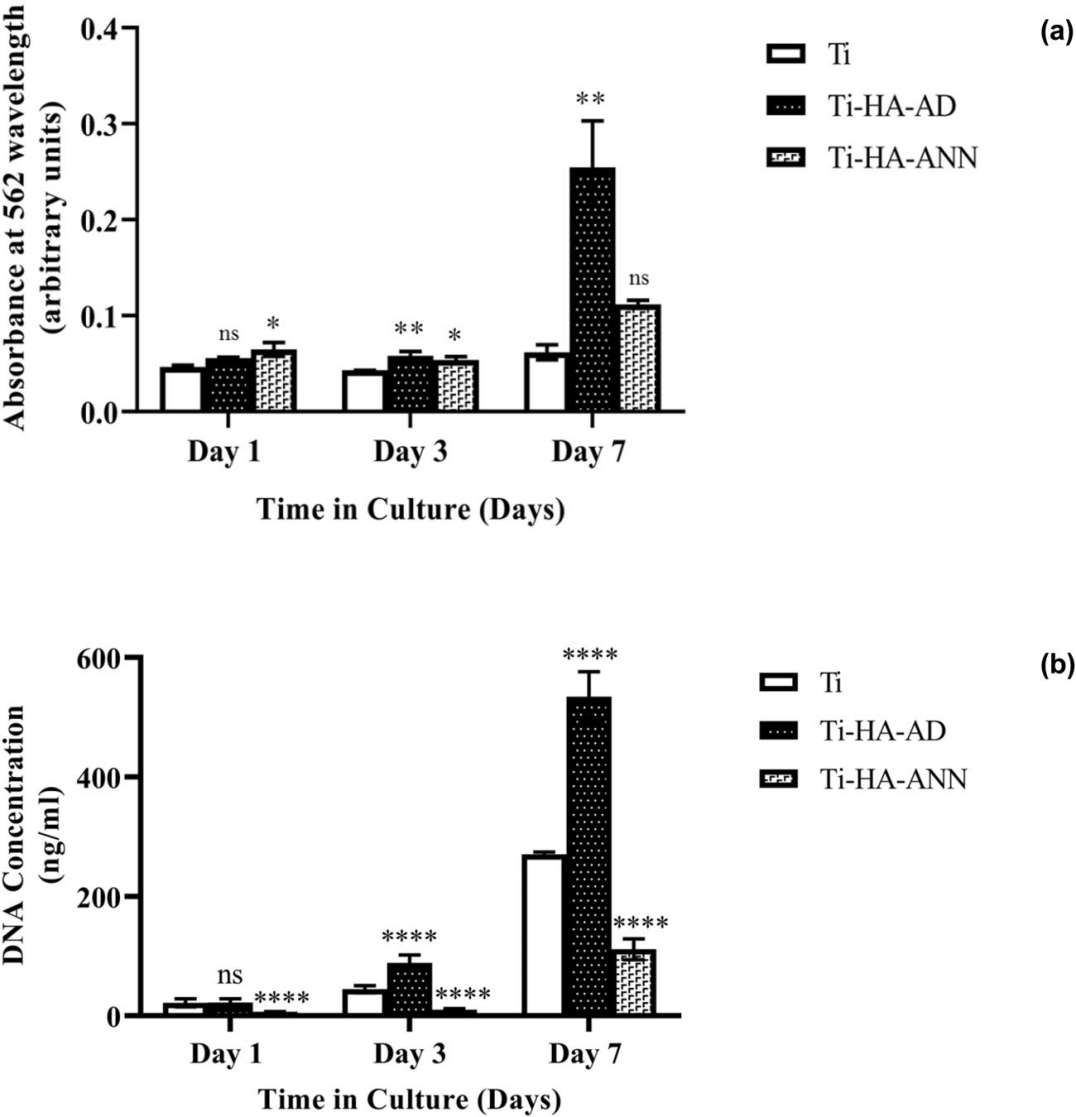
**a** MTT assay results representing cell metabolic activity for U-2 OS cells and **b** PG assay results representing DNA concentration for U-2 OS cells adhered to three types of Ti substrates (i) TI, (ii) TI-HA-AD and (iii) TI-HA-ANN, see legend, and cultured under standard conditions for 1, 3, and 7 days. Each substrate type was tested in triplicate. Error bars represent standard deviation. Any statistical significance between cells seeded and cultured on TI to those on two types of HA-coated Ti substrates (TI-HA-AD and TI-HA-ANN) represented by; not significant (ns) where *p* > 0.05, significant where *P* < 0.05. Increasing significance represented by increasing number of asterisks (*P < 0.05, **P < 0.01, ***P < 0.001 and ****P < 0.0001)

Two in vitro cell assays of three substrate types were performed at various timepoints following cell-seeding and culturing under standard conditions for up to 7 days. Each substrate type was tested in at least triplicate and results shown in Fig. 3, (a) represents MTT assay results and (b) represents PG assay results.

According to day 3 MTT assay results (Fig. 3a), cell metabolic activity was highest for TI-HA-AD (0.058 ± 0.004 au) followed by TI-HA-ANN (0.054 ± 0.004 au) and then by TI (0.043 ± 0.000). The same trend was demonstrated at day 7; TI-HA-AD (0.255 ± 0.049 au) followed by TI-HA-ANN (0.112 ± 0.004 au) and then by TI (0.062 ± 0.008 au). When compared to TI at same timepoints, MTT result for TI-HA-AD at day 3 and 7 demonstrated higher result of statistical difference (day 3 *p* = 0.0024 and day 7 *p* = 0.0019). When compared to TI at same timepoints, MTT result for TI-HA-ANN at day 3 demonstrated lower result of statistical difference (*p* = 0.00121) and while still lower at day 7 it was not statistically lower (*p* = 0.1588).

According to day 3 PG assay results (Fig. 3b), DNA concentration was highest for TI-HA-AD (88.64 ± 12.55 ng/ml) and contrary to MTT assay trend was followed by TI (44.55 ± 5.51 ng/ml) and then by TI-HA-ANN (10.43 ± 1.67 ng/ml). Once again at day 7 DNA concentration was highest for TI-HA-AD (534.35 ± 41.98 ng/ml) and contrary to MTT assay trend followed by TI (270.24 ± 3.41 ng/ml) and then by TI-HA-ANN (111.58 ± 1.64 ng/ml). When compared to TI at same timepoints DNA concentration for TI-HA-AD at day 3 and 7 was higher with statistical difference (*p* < 0.0001 for day 3 and 7). When compared to TI DNA concentration for TI-HA-ANN was statistically lower at all three timepoints (*p* < 0.0001 for day 1, 3, and 7).

The two in vitro cell assays gave comparable results with the general trend showing an increase in cell metabolic activity and DNA concentration for the three substrate types over time. For both in vitro cell assays TI-HA-AD had the highest results at day 1, 3, and 7. While TI-HA-AD demonstrated no statistical difference to that of TI at day 1 (MTT assay *p* = 0.1259, PG assay *p* = 0.9849) it did so at day 3 and 7 (day 3: MTT assay *p* = 0.0024, PG assay *p* < 0.0001 and day 7: MTT assay *p* = 0.0019, PG assay *p* < 0.0001).

### Single-cell analyses

#### Confocal-Raman spectroscopy

Biological characterisation was also performed using Confocal-Raman spectroscopy of cell-seeded quartz substrates from which cells were derived for the three Ti-based substrate types at day 7, 14, 21, and 28 and transferred onto quartz substrates, and three random single cells analysed at two cell locations, the nucleus and cytoplasm (not all spectra are shown). Each spectrum was repeated twelve times. It should be noted that it was difficult to locate three single cells on TI-HA-ANN at each timepoint suggesting that this substrate supported low cell numbers throughout the study.

**Fig. 4:**
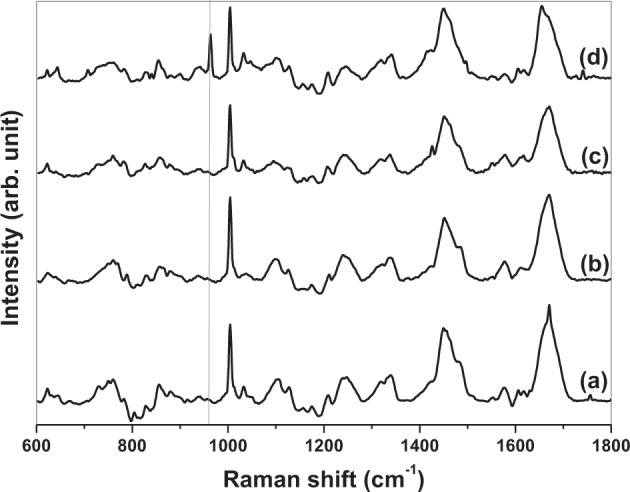
Stack of averaged processed Confocal-Raman spectra (*n* = 36) from the nuclei of three random single U-2 OS cells on quartz substrates. Cells derived from TI-HA-AD over 28 days in culture under standard conditions; **a** day 7, **b** day 14, **c** day 21, and **d** day 28. Grey solid line shows Raman peak at ~963 cm^−1^ at day 28 (strong)

**Fig. 5:**
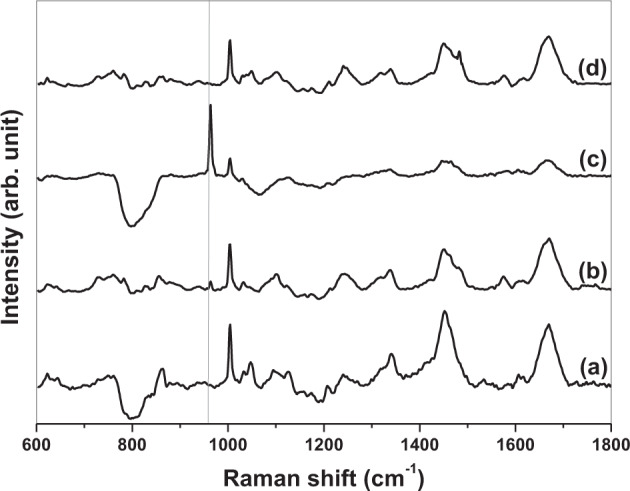
Stack of averaged processed Confocal-Raman spectra (*n* = 36) from the nuclei of three random single U-2 OS cells on quartz substrates. Cells derived from TI-HA-ANN over 28 days in culture under standard conditions; **a** day 7, **b** day 14, **c** day 21, and **d** day 28. Grey solid line shows Raman peak at ~963 cm^−1^ at day 14 (weak) and 21 (strong)

All spectra (Figs. 4, 5 and Supplementary Information Fig. 4) demonstrate much similarity and contain many Raman peaks within the expected spectral ranges for the main cellular constituents; biomolecules (Table 1). Figures show that all spectra regardless of (i) initial substrate type, (ii) cell location or, (iii) duration of culture time have three high intensity peaks at ~1004 cm^−1^ (phenylalanine), ~1454 cm^−1^ (C–H deformation) and ~1668 cm^−1^ (amide I) [21]. Another two peaks of less intensity are present for all spectra; ~1243 cm^−1^ (amide III) and ~1339 cm^−1^ (C–H bending) [21]. For all but one spectrum ~1004 cm^−1^ peak is the most intense (exception being Fig. 5c where it is ~963 cm^−1^ peak). An additional peak of interest exists for cells derived from TI-HA-AD and TI-HA-ANN; peak at ~963 cm^−1^ is present in both the nucleus (and cytoplasm, data not shown) within cells derived from TI-HA-AD at day 28 (Fig. 4) and in the nucleus (and cytoplasm, data not shown) within cells derived from TI-HA-ANN at day 21 (Fig. 5). As previously mentioned, peak at ~963 cm^−1^ is indicative of the P–O symmetric stretch (v_1_) within the PO_4_^3−^ present within HA [20]. In the main, Raman spectral signatures were similar at both cell locations for all substrate types at all timepoints however, there was observable loss of defined peaks and the presence of negative dips at ~770–900 cm^−1^ (namely 789 and 850 cm^−1^, which are indicative of DNA [22]) and ~1088–1102 cm^−1^ (indicative of nucleic acids, carbohydrates, and lipids, Table 1) for both cell locations at all timepoints for cells derived from TI-HA-ANN (Fig. 5 for the nucleus, cytoplasm data not shown). It is thought this was owing to the difficulty locating cells on this substrate, as also reported in Section “SEM”.

**Table 1:** Typical Raman spectral ranges for a single cell and the biomolecules corresponding to that range [21]

Spectral wavenumber range (cm^−1^)	Corresponding biomolecule
718–790	Lipids and nucleic acids
815–830	RNA and proteins
1088–1102	Nucleic acids, carbohydrates, and lipids
1243–1348	Nucleic acids, proteins, and lipids
1550–1580	Nucleic acids

**Fig. 6:**
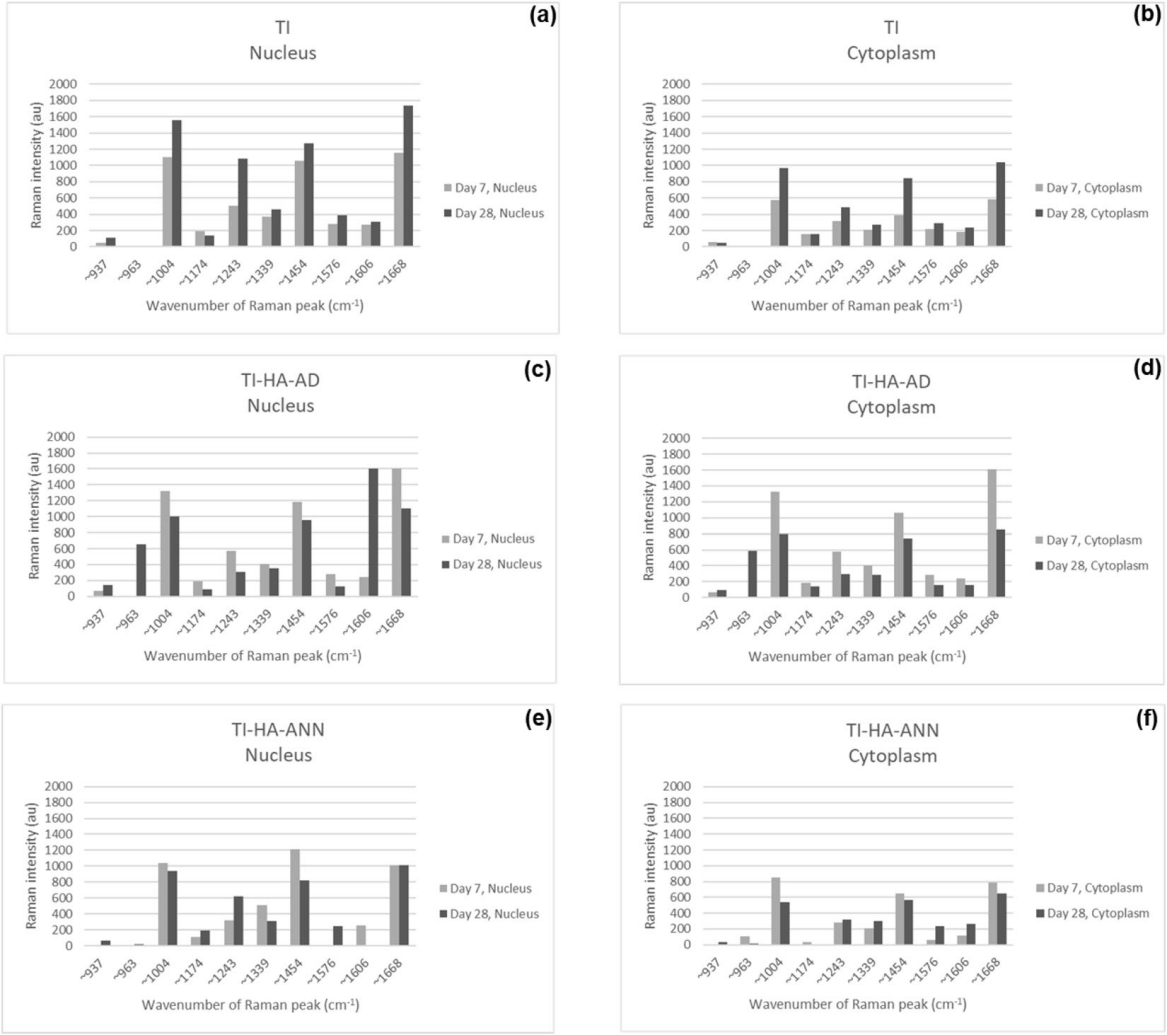
Averaged processed Raman intensities (*n* = 36) as a bar chart for the main Raman peaks (937–1668 cm^−1^) detected in the nucleus (**a**, **c**, **e**) and cytoplasm (**b**, **d**, **f**) for three random single U-2 OS cells on quartz substrates after 7 (light grey bars) and 28 (dark grey bars) days in culture under standard conditions. Cells derived from; **a**, **b** TI, **c**, **d** TI-HA-AD and **e**, **f** TI-HA-ANN. Where Raman intensity is 0 au no Raman signal was detected

To observe any notable changes over culture time in Raman intensities for the main peaks highlighted by Figs. 4 and 5 (and Supplementary Information Fig. 4), Raman data for the first and last timepoints (day 7 and 28, respectively) were plotted. Figure 6 demonstrates averaged processed Raman intensities for the main Raman peaks (937–1668 cm^−1^) detected in the nucleus and cytoplasm for the three substrate types, TI (Fig. 6a, b), TI-HA-AD (Fig. 6c, d), and TI-HA-ANN (Fig. 6e, f), respectively. The general trend for peak intensities for all three substrate types is that they are higher in the nucleus than in the cytoplasm. In general, for the uncoated substrate, TI, peak intensities increased for both cell locations from day 7 to day 28. Highly similar spectral profiles were obtained for both cell locations for both timepoints. More notable changes in spectral profiles were obtained for HA-coated substrates, TI-HA-AD and TI-HA-ANN. A peak at ~963 cm^−1^ is present in both the nucleus and cytoplasm for cells derived from TI-HA-AD at day 28 (Fig. 6d). This peak was not present at day 7 (Fig. 6c). As previously mentioned, peak at ~963 cm^−1^ is indicative of the P–O symmetric stretch (v_1_) within the PO_4_^3−^ present within HA [20]. Intensity of peak at ~1606 cm^−1^ (indicative of adenine vibration in DNA [23]) detected in the nucleus for cells derived from TI-HA-AD at day 7 increased six-fold from day 7 to day 28 (Fig. 6c) however, there was minimal change detected in the cytoplasm over the same time period (Fig. 6d). Peak intensities detected in both cell locations for cells derived from TI-HA-ANN were lower than those from TI or TI-HA-AD, with intensities no higher than ~1250 au regardless of culture time (Fig. 6e, f) while those from TI and TI-HA-AD reached ~1750 au by day 28 (Fig. 6a, b and c, d, respectively). As previously reported, there was difficulty locating cells derived from TI-HA-ANN as reported in Section “SEM”. Intensity of peak at ~1243 cm^−1^ (indicative of amide III [21]) detected in the nucleus for cells derived from TI-HA-ANN at day 7 increased two-fold from day 7 to day 28 (Fig. 6e) however, there was minimal change detected in the cytoplasm over the same time period (Fig. 6f). Peak at ~1576 cm^−1^ was not detected in the nucleus for cells derived from TI-HA-ANN at day 7 however, it was at day 28 (Fig. 6e). Peak at ~1576 cm^−1^ (indicative of DNA, Table 1) was detected in the cytoplasm for cells derived from TI-HA-ANN at day 7 and increased four-fold by day 28 (Fig. 6f).

#### SEM-EDX

Additional biological characterisation was performed using SEM-EDX to further characterise areas of interest within some cell-seeded quartz samples whereby single U-2 OS cells derived from TI-HA-AD and TI-HA-ANN substrates gave strong peaks at ~963 cm^−1^ by Raman spectroscopy after 21 and 28 days in culture under standard conditions. Three single cells on these samples were analysed. It should be noted that it was difficult to locate cells derived from TI-HA-ANN, as reported in Section “SEM”.

**Fig. 7:**
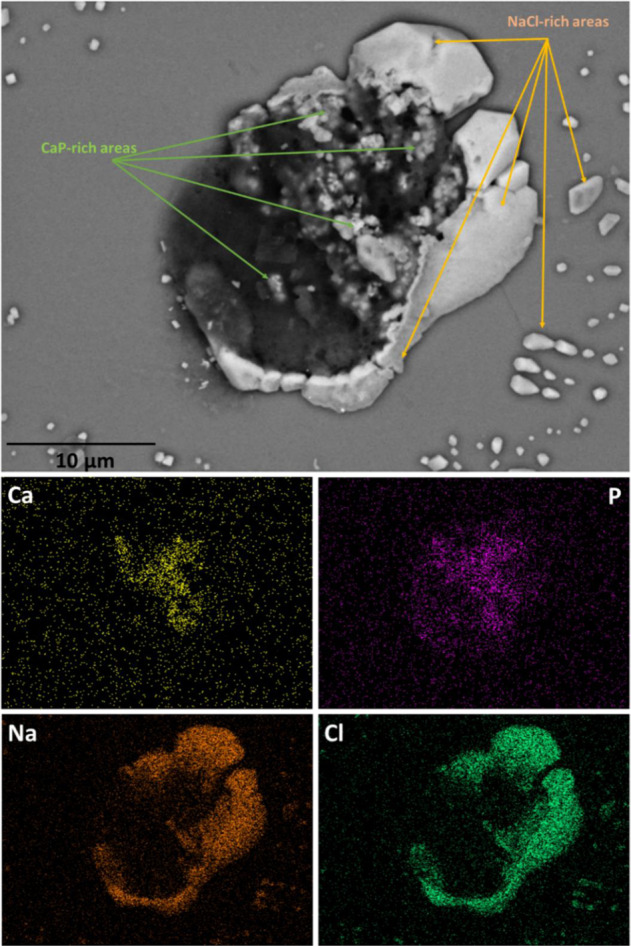
SEM image (main image) along with corresponding elemental maps (Ca, P, Na, and Cl) from quartz substrate for single U-2 OS cell derived from TI-HA-AD after 28 days in culture under standard conditions. Scale bar represents 10 µm distance within main image

Figure 7 shows SEM image (main image) along with corresponding elemental maps from quartz substrate for single U-2 OS cell derived from TI-HA-AD after 28 days in culture under standard conditions. The C map corresponds to organic matter within the cell and, Si and O maps correspond to quartz substrate. Presence of Na and Cl, as indicated by Na and Cl maps, represent residual salt from use of PBS in the biological methods. The P map represents various parts of the cells with P content such as, cell membrane, cell organelles and genetic material. C and P were the most abundant elements present within the cell. The Ca and P maps show elemental correlation in the same locations within the cell indicating presence of CaP, which in turn suggests presence of intracellular HA, an indicator of bone cell maturation [24]. Additional elemental maps corresponding to Si, O, and C are shown in Supplementary Information Fig. 6. It should be noted that for single U-2 OS cell derived from TI, no Ca was detected, the absence of Ca in cells indicates the absence of HA within cells derived from TI (Supplementary Information Fig. 5).

**Fig. 8:**
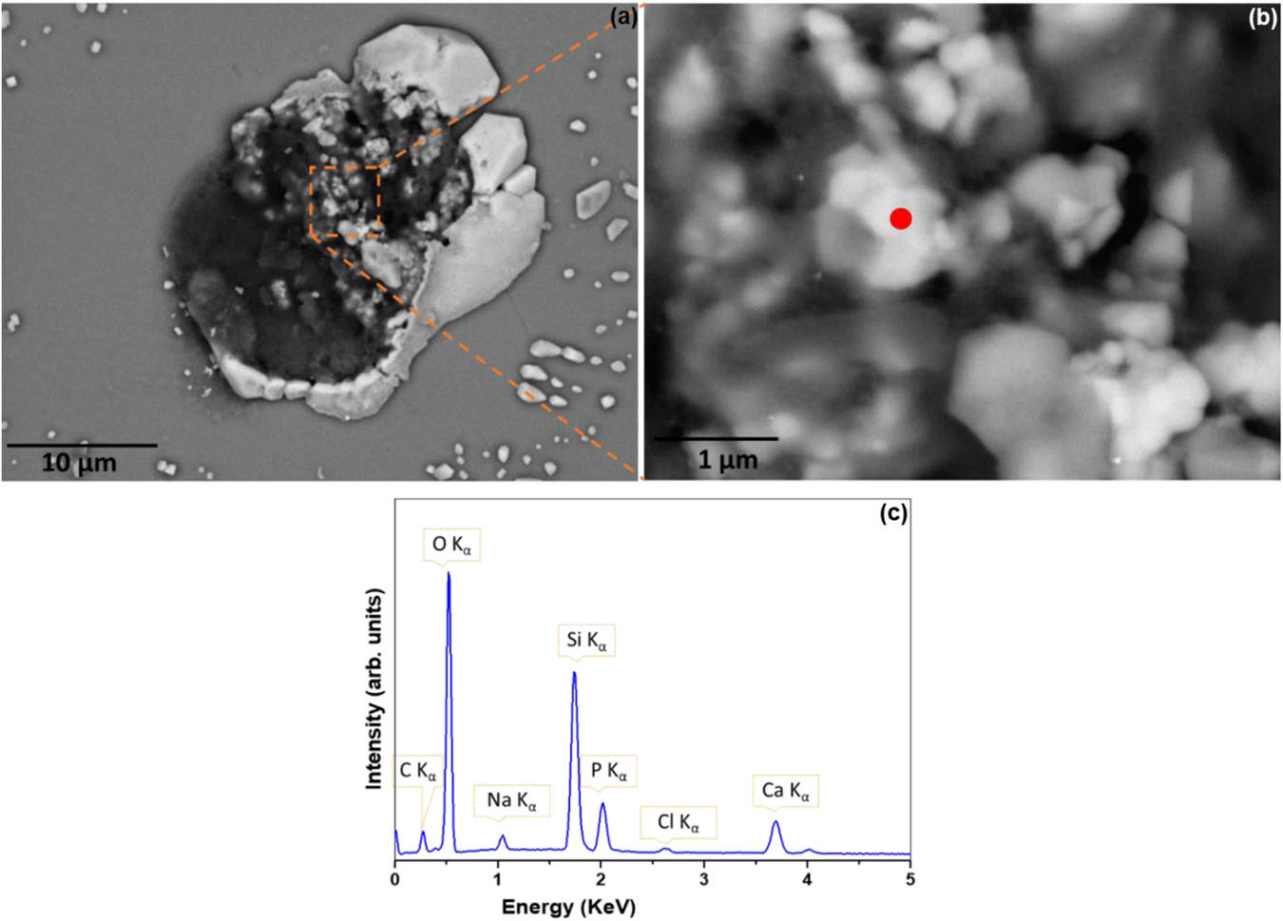
**a** SEM image (main image) along with additional SEM-EDX information from quartz substrate for single U-2 OS cell derived from TI-HA-AD after 28 days in culture under standard conditions, **b** zoomed-in SEM image from (**a**) and (**c**) point spectra corresponding to red dot within (**b**). Scale bar represents 10 µm distance within main image

To confirm intercellular HA, point spectra at different locations within the cell (of Fig. 8) were acquired. A zoomed-in region is shown in Fig. 8b and representative spectra corresponding to the red dot shown in Fig. 8c. Calculation from five different spectra calculated the Ca/P ratio as 1.63 ± 0.5, a substantial increase on that calculated for synthetic HA coating (Ca/P ratio by XPS was 0.8, Ca/P ratio by SEM-EDX was 1.28, Section “HA coating”), suggesting precipitation of natural HA within the cell. No extracellular HA was detected. HA particles were 100–400 nm in size, with average size 230 nm, and along with their size, their geometry was different from that of the relatively rounded and clustered particles of the HA coating (Supplementary Information Fig. 1b and Section “HA coating”) with many particles showing sharp edges and crystalline facets (Fig. 8b).

**Fig. 9:**
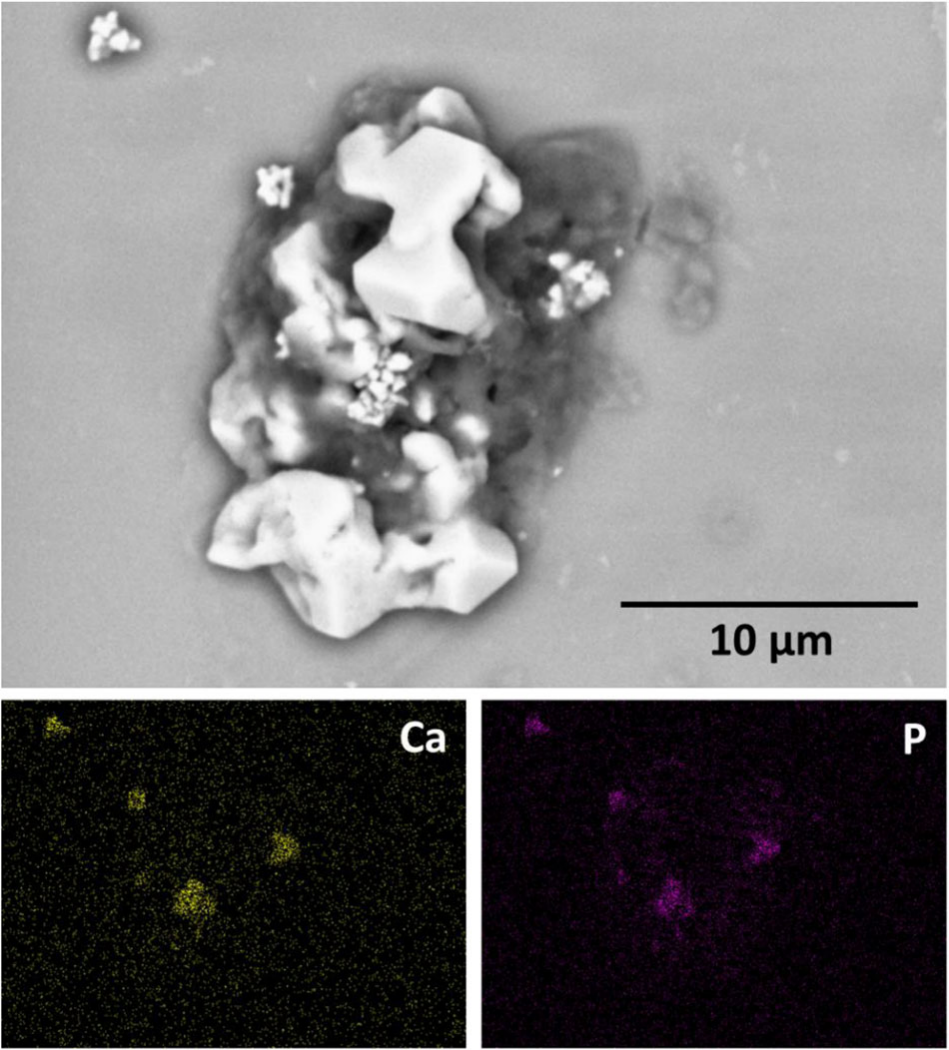
SEM image (main image) along with corresponding elemental maps (Ca and P) from quartz substrate for single U-2 OS cell derived from TI-HA-ANN after 21 days in culture under standard conditions. Scale bar represents 10 µm distance within main image

Figure 9 shows SEM image (main image) along with corresponding elemental maps from quartz substrate for single U-2 OS cell derived from TI-HA-ANN after 21 days in culture under standard conditions. Additional elemental maps corresponding to Na, Cl, O, Si, and C are shown in Supplementary Information Fig. 7. Once again, presence of Na and Cl, as indicated by Na and Cl maps, represent residual salt, and in this case a heavy precipitation of salt from use of PBS in the biological methods. The Ca and P maps show elemental correlation in the same locations both within the cell and outside the cell, indicating presence of CaP and possible intra- and extracellular HA. While intra- and extracellular HA show similar morphology, their size differs; intracellular HA had average size of 280 nm (size range 100–550 nm) while that of extracellular HA was ~300 nm with no particle less than 200 nm.

**Fig. 10:**
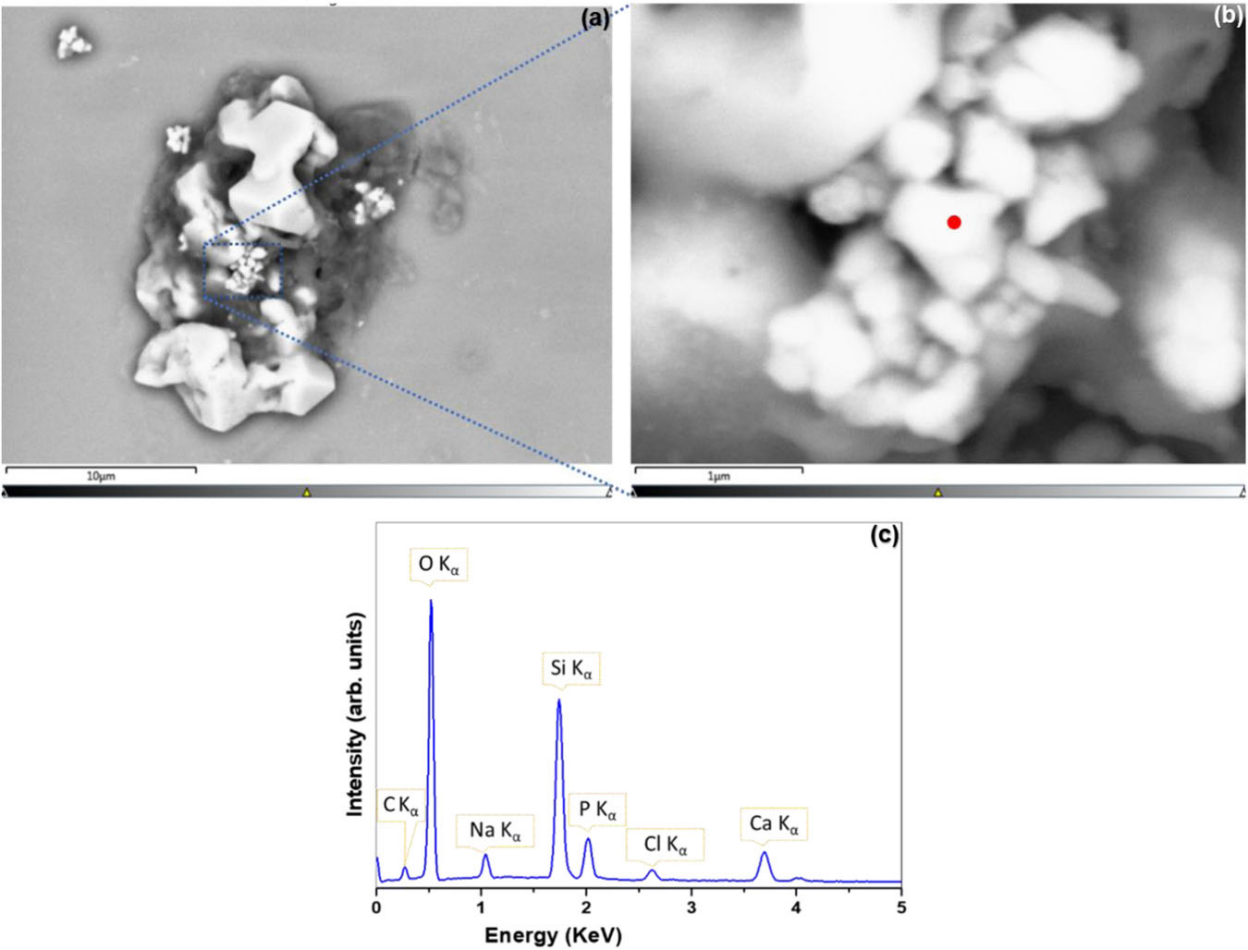
**a** SEM image (main image) along with additional SEM-EDX information from quartz substrate for single U-2 OS cell derived from TI-HA-ANN after 21 days in culture under standard conditions, **b** zoomed-in SEM image from (**a**) and (**c**) point spectra corresponding to red dot within (**b**). Scale bar represents 10 µm distance within main image

To confirm the source and possible migration of intracellular HA, point spectra at different locations within the cell (of Fig. 9) were acquired. The zoomed-in region from Fig. 10a is shown in Fig. 10b and representative spectra corresponding to the red dot shown in Fig. 10c. The spectrum has peaks for C, O, P, Ca, Na and Cl, as expected. Once again, Ca/P ratios (1.68 ± 0.3) were higher than those of synthetic HA coating and similar, suggesting intracellular precipitation of HA. In addition, in this case, intracellular HA migrated outwardly from the cell hence, its presence in extracellular regions also.

It should be noted that for the majority of single U-2 OS cell derived from TI-HA-ANN after 28 days in culture under standard conditions the Si and O signals corresponding to quartz substrate were not as prominent as previous quartz substrates and that while Ca signals were detected there was no marked P signal (Supplementary Information Figs. 8, 9). Presence of Ca but not P indicates the formation of a Ca-based compound, other than HA, with significant Ca and O signals suggesting that the Ca-based compound is CaO (particle size 20–400 nm, Supplementary Information Fig. 9). Intracellular HA was only present in 1–2 cells; blue dotted box within Supplementary Information Fig. 10 as confirmed by point spectra corresponding to the red dot within Supplementary Information Fig. 10b where the Ca/P ratio was 1.67, similar to previous results for intracellular HA.

## Discussion

Initially in this study, a layer of HA was mechanically deposited onto substrates of Ti using RF magnetron sputtering deposition with subsequent chemical characterisation techniques, XPS, SEM, SEM-EDX and Raman spectroscopy, confirming successful coating. Analysis by XPS and SEM-EDX yielded differing results for Ca/P ratios (0.8 and 1.28, respectively). The disparity could be due to several variables; (i) the coating characteristics, (ii) the employed sputtering conditions and (iii) the associated depth of analysis for each technique. XPS can analyse a depth of ~5–10 nm (under the conditions employed in this study) and SEM-EDX can analyse a depth of ~0.9 µm. Both techniques confirm the presence of both Ca and P on the surface but the application of dry pressed HA targets in this study can lead to re-sputtering of the HA coating, resulting in a lower-than-expected Ca/P ratio and possible disparities between methods [25–27]. In addition, said characterisation techniques provided information on coating uniformity on the Ti substrate; no Ti 2p signals were obtained by XPS (Fig. 1 and Section “HA coating”) suggesting a homogeneous coating while Ti signals of small magnitude from SEM-EDX suggested otherwise (Supplementary Information Fig. 1d and Section “HA coating”). SEM-EDX showed synthetic HA particles were relatively round with average particle size 372 nm.

Synthetic HA is widely used in biomaterials designed for bone implantation as it has been widely reported to promote bone regeneration [28–30]. It is theorised that its bone regeneration potential is enhanced in crystalline form due to its reduced solubility and longer presence as a solid-state interface for interaction with cells. In this study, the bioactive potential of re-crystallised HA was further explored by annealing HA-coated substrates (at 500 °C) (TI-HA-ANN) while leaving half the samples untouched, as-deposited (TI-HA-AD). As previously reported, heating under such conditions induces phase composition changes converting Ca/P from amorphous solid to crystalline solid [31, 32]. Coated substrates, TI-HA-AD and TI-HA-ANN, were exposed to medium under standard conditions for 28 days and then subjected to further chemical characterisation by Raman spectroscopy with day 0 and 28 Raman spectra compared (Supplementary Information Figs. 2, 3, respectively). Surprisingly, there were no observable differences; Raman peaks indicative of HA (~960 cm^−1^ [20]) were still present (and of similar intensity to those of day 0) demonstrating the continued presence of HA coating after 28 days in solution, regardless of the ‘as deposited’ or ‘annealed’ HA state. In addition, SEM images taken at day 28 provide further evidence that the HA coating, regardless of phase composition, was still present 28 days after exposure to medium (Fig. 2b for TI-HA-AD and Fig. 2c for TI-HA-ANN). These images show a ‘break’ in HA coating exposing the underlying Ti substrate. Uniform coverage of relatively round and clustered particles seen in Supplementary Information Fig. 1b is no longer present; a flatter, ‘flake-like’ and layered appearance is observed.

The biological characterisation of TI-HA-AD and TI-HA-ANN was performed using two in vitro cell assays (at day 1, 3, and 7) and Confocal-Raman spectroscopy (at day 7, 14, 21, and 28). For all three methods, results were compared to those from uncoated substrate (TI) hence, TI was the control. In vitro cell assay results were comparable, demonstrating a general trend of increasing measurable output (cell metabolic activity for MTT assay and DNA concentration for PG assay) with culture time for all three substrates (Fig. 3a for MTT assay and Fig. 3b for PG assay). Given reports in the literature on enhanced bioactivity of synthetic HA and its theorised enhanced bioactivity in crystalline form [4], it was anticipated that the annealed HA-coated substrate would render the highest results for both methods to reflect promoted cell adhesion and proliferation. As-deposited HA-coated substrate (TI-HA-AD) rendered the highest results for both methods for all timepoints over that of TI (statistically higher at day 3 and 7) and surprisingly also over that of the annealed HA-coated substrate (TI-HA-ANN). A less surprising result was the lack of consensus between the two in vitro cell assays for TI and TI-HA-ANN; a higher result obtained by one method but not with the other (results for TI-HA-ANN were higher than TI for all timepoints by MTT assay, statistically higher at day 1 and 3 albeit statistically lower than TI for all timepoints by PG assay). A lack of consensus may be attributable to the differences in what is measured by each assay; MTT assay measures active metabolism of viable cells whereas, PG assay measures DNA concentration. In addition, issues with MTT assay such as, its destructive method and lack of specificity, accuracy, and reproducibility have been reported [33, 34]. Recent years have seen more use of DNA quantification assays such as, PG assay, because their higher specificity and sensitivity better represents cell viability, adhesion and proliferation [35, 36]. Overall, consensus from the two in vitro cell assays was that the as-deposited HA-coated substrate was able to ‘better’ support cells, in this case U-2 OSs, than the uncoated Ti substrate, suggesting that the presence of as-deposited HA on a biomaterial enhances biomaterial bioactivity more than annealed HA.

SEM images collected from three cell-seeded substrate types at day 28 provided qualitative information on surface topography and cell morphology (where cells were present). Cells were present on all three random locations on TI and TI-HA-AD (Fig. 2a, b, respectively) but not on TI-HA-ANN (Fig. 2c). Further locations were analysed on TI-HA-ANN but no cells were found. Cells on TI and TI-HA-AD demonstrated typical morphology for adhered U-2 OSs; epithelial adherent morphology and cells of 25–50 µm in diameter with a large cell body, as expected for U-2 OSs [19]. Cell morphology is similar between cells shown for TI and TI-HA-AD however, it could be suggested that cells on TI have more pronounced cell bodies while those on TI-HA-AD have flatter, more elongated appearance (Fig. 2a, b, respectively). This would be a reasonable suggestion given their differing surface topographies. Adhering cells will change shape to accommodate such surface changes and to ‘best anchor’ themselves on the surface. It would appear from Fig. 2a that cells would have less topography/roughness to interrogate given the unidirectional linear striations as expected for a bare metal surface, possibly causing cells to ‘stretch less’ and hence, their cell bodies to be more pronounced on the surface. HA-coated Ti (Fig. 2b) appears to have uniform coverage of relatively rounded and clustered particles, with more uneven topography/roughness for cells to ‘stretch out’ to reach randomly orientated nano-sized pores and pits for secure anchorage thereby, becoming ‘flatter’ in morphology. The leading theory reported in the literature is that cells have enhanced proliferation on rough surfaces [37]. Comparing images in Fig. 2a, b suggests that more cells adhered to TI than to TI-HA-AD. This would be contrary to the anticipated result given earlier culture time results from in vitro cell assays that demonstrated TI-HA-AD to be significantly ‘better’ than TI and additional to this, the roughness offered by HA-coated surface. With regards the absence of cells on TI-HA-ANN, this result is not surprising given earlier culture time results from in vitro cell assays, especially if PG assay results are deemed to be more accurate, as previously discussed, as TI-HA-ANN was significantly ‘worse’ than TI. These results highlight the importance of optimal and if possible, enhanced cell adhesion, for promotion of subsequent cell processes allowing for continued cell proliferation followed by cell maturation as later culture times [38].

Biological characterisation continued using Confocal-Raman spectroscopy. It was deemed unfeasible to perform Raman analysis on cell-seeded TI substrates due to background Raman spectra owing to HA and Ti that would mask or diminish any spectra owing to any cells present. To overcome this, at each timepoint adhered cells were trypsinised from Ti-based substrates, placed on quartz (a ‘Raman-compatible’ substrate with minimal Raman effect) and chemically fixed ready for Raman analysis. Average processed spectra demonstrate much similarity and typify expected protein/lipid dominated Raman signature for cells (Figs. 4, 5 and Supplementary Information Fig. 4) [21]. All spectra display many of the peaks attributable to the main cellular constituent, biomolecules (Table 1) and display the same three high intensity peaks (~1004 cm^−1^ (phenylalanine), ~1454 cm^−1^ (C–H deformation) and ~1668 cm^−1^ (amide I)) and two further lower intensity peaks (~1243 cm^−1^ (amide III) and ~1339 cm^−1^ (C–H bending)) [21]. This spectral chemical signature was generally present at both cell locations (nucleus and cytoplasm, not all data shown) at all timepoints (day 7, 14, 21, and 28) for all three substrate types (TI, TI-HA-AD and TI-HA-ANN) demonstrating that cells were viable and able to adhere and proliferate for the study’s duration (28 days). On a qualitative level, Raman results support in vitro cell assay results; cells were viable and able to adhere and proliferate on all three substrate types for 7 days albeit at varying degrees. In addition, Raman results demonstrate important information; that this continued for a further 21 days. It was noted that there was an observable loss of defined peaks for cells derived from TI-HA-ANN over 28 days in culture (Fig. 5), namely those associated with nucleic acids, carbohydrates and lipids, indicating compromised cell health. Indeed, in this study it was widely reported that these cells gave ‘poor’ in vitro cell assay results (at day 1, 3, and 7) and were difficult to locate for SEM analysis (at day 28), indicating low levels of cell adhesion and proliferation, and the Raman spectroscopy results support this indication. Raman results provide further evidence that the strong peak attributable to phenylalanine (~1004 cm^−1^) has value as a cellular ‘biomarker’ [39–41]. It was the most intense peak for all but one spectrum (exception being Fig. 5 where it was ~963 cm^−1^ peak). In fact, as previously mentioned, 963 cm^−1^ is indicative of the P–O symmetric stretch (v_1_) within the PO_4_^3−^ present within HA [20] and is the most intense Raman peak in bone [19]. In general, Raman intensities for the main peaks, as described above, gave a higher intensity in the nucleus than in the cytoplasm (Fig. 6). This is an expected result given the enrichment of nucleic acids within the nucleus; Raman peaks indicative of nucleic acids span the wavenumber range of interest 600–1800 cm^−1^ (Table 1). Spectral profiles for cells derived from TI demonstrated a high degree of similarity albeit intensities increased from day 7 to day 28 (Fig. 6a, b), an increase in intensities for the same peaks over time (or concentration of the same biomolecules over time) is indicative of cell proliferation as opposed to cell maturation (there was no evidence of cell maturation over culture time for these cells from Raman spectroscopy or SEM-EDX (Supplementary Information Fig. 5). More notable changes in spectral profiles were obtained for HA-coated substrates, TI-HA-AD and TI-HA-ANN. Peak at ~963 cm^−1^ was detected (with same intensity) in both the nucleus and cytoplasm for cells derived from TI-HA-AD at day 28 (Fig. 6d) but not present at day 7 (Fig. 6c). Its presence is indicative of cell maturation over culture time and the formation of natural HA within de novo bone (and further supported with later evidence of intracellular HA in the same cells at day 28 by SEM-EDX). It is likely that this maturation process explains the large increase in intensity of a peak at 1606 cm^−1^ indicative of the adenine vibration in DNA detected in the nucleus from day 7 to 28, Fig. 6c and d, respectively. While the same evidence of cell maturation existed in cells derived from TI-HA-ANN, it appeared at an earlier timepoint, day 21, by Raman spectroscopy and SEM-EDX (Fig. 5 and 9, 10, respectively). These cells maturating more speedily than those derived from TI-HA-AD may explain intensity increases in Raman peaks indicative of proteins and nucleic acids observed within the nucleus of the same cells from day 7 to day 28 (1243 cm^−1^ indicative of amide III [21] and 1576 cm^−1^ indicative of DNA/RNA [10], Fig. 6e, f). Lower Raman intensities for all peaks for both cell locations over culture time were noted for cells derived from TI-HA-ANN in comparison to those from TI or TI-HA-AD, with intensities decreasing further from day 7 to 28. It is a plausible suggestion that spectral profiles for cells derived from TI-HA-ANN demonstrate the opposite trend to those from TI (Fig. 6a, b) and in doing so, showcase cell maturation as opposed to cell proliferation. As previously mentioned, no cells were found on the TI-HA-ANN substrate (at day 28) during SEM analysis (Section “SEM”) and it was difficult to locate the same cells on the quartz substrates (at day 7, 14, 21, and 28) for Raman analysis (Section “Confocal-Raman spectroscopy”). The absence/lack of cells is a surprising result given that the two in vitro cell assays, MTT and PG, provide evidence of cell viability, adhesion, and proliferation (through cell metabolism and cell quantity data, respectively, Fig. 3) on the TI-HA-ANN substrate, albeit less than that on TI-HA-AD, and similar to that on TI. It can only be surmised that the viable cells present at day 7 were not in a healthy cell state as, following trypsinisation onto quartz substrates for Raman analysis, very few cells could be located (at day 7) and less again at the later timepoints (at day 14, 21, and 28) hence, the absence of cells for SEM analysis at day 28. It would appear that conditions were not optimal when cells were exposed to the TI-HA-ANN substrate and that cell maturation was promoted over cell proliferation at the detriment of the few cells adhered on this surface.

CaP ceramics are usually described by their Ca/P atomic ratio [42]. As previously mentioned, stoichiometric HA has Ca:P ratio of 1.67 [43]. In this study, a CaP ceramic (synthetic HA) was coated onto Ti and shown to have Ca/P ratio of 0.8 by XPS (Fig. 1) and 1.28 by SEM-EDX (Supplementary Information Fig. 1d). SEM-EDX also demonstrated a CaP compound within cells derived from HA-coated substrates at later timepoints (day 28 for TI-HA-AD and day 21 for TI-HA-ANN) with Ca/P ratio of 1.63 ± 0.5 for TI-HA-AD and 1.68 ± 0.3 for TI-HA-ANN. Similarity of Ca/P ratio for this CaP compound to that of stoichiometric HA (1.67) suggests that these cells at later stages of culture were producing intracellular HA. Not only was Ca/P ratio an indicator of a “difference” between the HA coating and intracellular HA but their geometry was also; average HA coating particle was 372 nm (particles size range 280–520 nm) (Supplementary Information Fig. 1e) and relatively round in shape (Supplementary Information Fig. 1c, e) while that of intracellular HA and extracellular HA were smaller (intracellular: average particle size 280 nm, particle size range 100–550 nm, extracellular: average particle size ~300 nm with no particle less than 200 nm), more irregular in shape and displayed sharp, pointed edges (Figs. 8b and 10a, b).

Interestingly, SEM-EDX also showed the same CaP compound on the periphery of cells derived from TI-HA-ANN suggesting that extracellular HA had migrated outwardly from within the cells (Fig. 10a). In fact, Fig. 10a shows HA particles crossing the cell membrane. Intracellular HA was detected in cells derived from TI-HA-ANN 7 days before those from TI-HA-AD (day 21 and 28, respectively). While SEM-EDX detected intracellular HA in cells derived from TI-HA-ANN after 28 days it was only present in 1–2 cells (Supplementary Information Fig. 10) which may explain the absence of expected ~963 cm^−1^ Raman peak at day 28 (Fig. 5). As previously mentioned and discussed, results suggest that cells derived from TI-HA-ANN were low in numbers (if not absent) at all timepoints (Section “SEM”) and that cell health was compromised (Section “Confocal-Raman spectroscopy”) however, Raman spectroscopy and SEM-EDX results suggest that those that were present were mineralising and forming natural HA (a major bone constituent) and doing so earlier than those from TI-HA-AD.

An interesting SEM-EDX result was the presence of a Ca-based compound, presumed to be CaO by interpretation of Supplementary Information Fig. 9c, present in cells derived from TI-HA-ANN at day 28. Ca/P ratios can range from 0.5 to 2 and can be synthesised by mixing Ca and P solutions under acid or alkaline conditions [44]. When Ca/P ratio is lower than 1.67, β-tricalcium phosphate (TCP) and other phases such as, tetracalcium phosphate (TTCP) will exist within HA, and if higher than 1.67, CaO will exist [44]. Intracellular HA within cells derived from TI-HA-ANN had Ca/P ratio of 1.68 ± 0.3 (Fig. 10c); this higher Ca/P ratio may explain the presence of CaO within these cells and its absence within cells with intracellular HA of lower Ca/P ratio (1.63 ± 0.5, TI-HA-AD at day 28, Fig. 8c).

Overall in this study, the single-cell derived Confocal-Raman peak at ~963 cm^−1^ proved to be the most interesting, not only due to its presence but also in identifying its origin. As previously mentioned, ~963 cm^−1^ was not observed in the nucleus or cytoplasm of cells derived from TI at any timepoint but was a strong peak in the (i) nucleus and cytoplasm of cells derived from TI-HA-AD at day 28 and (ii) nucleus and cytoplasm of cells derived from TI-HA-ANN at day 21. It was decided to use SEM-EDX to further characterise cell-seeded quartz substrates. The aim was to use SEM to further image cells derived from HA-coated substrates and then to use EDX to determine chemical composition of any intracellular HA present in an attempt to confirm its origin; whether it was residual HA coating or native bone formation. In support of SEM-EDX results (i) no intracellular HA was present for cells derived from TI, Supplementary Information Fig. 5 (supported by absence of Raman peak indicative of HA, Supplementary Information Fig. 4), (ii) intracellular HA was detected in cells derived from TI-HA-AD at day 28, Figs. 7, 8 (supported by Raman peak indicative of HA, Fig. 4) and (iii) intracellular HA was detected in cells derived from TI-HA-ANN at day 21, Figs. 9 and 10 (supported by Raman peak indicative of HA, Fig. 5). While calcification is typically extracellular, Azari et al. reported on intracellular HA precipitation within diseased cell lines, like that used within this study, U-2 OS (from an osteosarcoma), and might lead to pathological calcification of soft tissues [24]. As described above, additional characterisation of HA particle size, shape and Ca/P ratio by SEM-EDX, supports the suggestion that the Raman peak indicative of HA at later culture times (day 21 and 28) represents cell maturation. No such peak was found in cells derived from TI adding support for HA’s bioactive properties which was also supported by in vitro cell assays and Raman spectroscopy. Bone cell maturation is not common under non-osteogenic conditions. McManus et al. had evidence from phase contrast micrographs of discrete mineral deposition within extracellular matrix of monolayer of U-2 OS cells 14 days after seeding and culturing on calcium fluoride substrates under non-osteogenic conditions and from alizarin red staining and quantification evidence of extensive mineralisation after 28 days [19]. In this study Raman spectroscopy and SEM-EDX demonstrated evidence of bone cell maturation under non-osteogenic conditions as early as 21 days after seeding and culturing on HA-coated TI substrates, with SEM-EDX showing intracellular HA migrating and ready to cross the cell membrane to the extracellular matrix.

## Conclusions

In this study Ti, HA and Raman spectroscopy were employed to provide new and additional support for their use in analysing osteoblast cells in TERM applications. Results demonstrated; (i) further support for adding HA to a biomaterial due to its bioactive worth whereby it promotes cell viability, adhesion and proliferation during the early culture days, as determined by two traditional cell assay methods and promotes cell maturation as the culture time progresses, as determined by the less traditional biological characterisation technique, Raman spectroscopy, (ii) HA coating (both as-deposited and annealed) remained on the substrates in solution for up to 28 days and (iii) Raman spectroscopy can be employed to monitor the cellular response, in particular cell maturation towards de novo bone, of single bone cells, U-2 OSs, after continued exposure to HA-coated Ti under non-osteogenic conditions via a rapid label-free method capable of sequential testing.

## Supplementary Information

Supplementary Information
