# Supplementary material for: Direct monitoring of single-cell response to biomaterials by Raman spectroscopy
Source: J Mater Sci Mater Med. 2021 Dec 4;32(12):148. doi: 10.1007/s10856-021-06624-5 (PMC8643295; doi:10.1007/s10856-021-06624-5)
Supplement: Supplementary file 1 — Supplementary Information [file 10856_2021_6624_MOESM1_ESM.docx]

**Journal of Materials Science: Materials in Medicine**

**Direct monitoring of single-cell response to biomaterials by Raman spectroscopy**

Mary Josephine McIvor^1^*, Preetam K. Sharma^1,2^, Catherine E. Birt^1^, Hayley McDowell^1^, Shannon Wilson^1^, Stephen McKillop^1^, Jonathan G. Acheson^1^, Adrian R. Boyd^1^, Brian J. Meenan^1^

**Affiliation^1^:** Nanotechnology and Integrated Bioengineering Centre (NIBEC), School of Engineering, University of Ulster, Shore Road, Newtownabbey, Co. Antrim, BT37 0QB. Northern Ireland (UK).

**Affiliation^2^:** Department of Chemical Engineering, Loughborough University, Loughborough, LE11 3TU. England (UK).

**Corresponding author:** Mary Josephine McIvor, Nanotechnology and Integrated Bioengineering Centre (NIBEC), School of Engineering, University of Ulster, Shore Road, Newtownabbey, Co. Antrim, BT37 0QB. Northern Ireland (UK). Tel: +44 (0)28 90 366319. Fax: +44(0)28 90 366863. e-mail: [mj.mcivor@ulster.ac.uk](mailto:mj.mcivor@ulster.ac.uk) [ORCID ID: 0000-0002-2451-9304](https://orcid.org/0000-0002-2451-9304).

**Supplementary Information (one figure per page)**

**(b)**

**(a)**


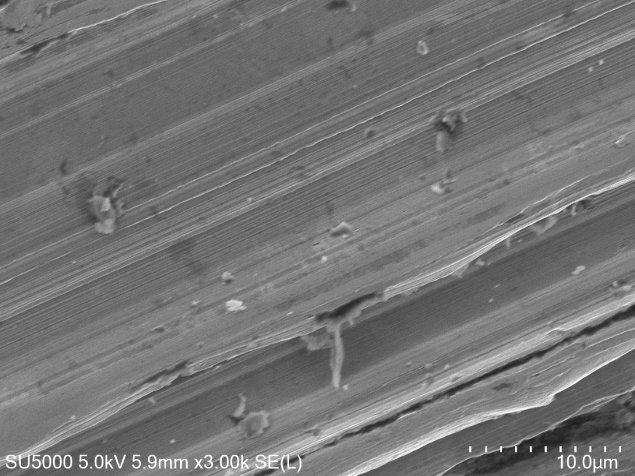

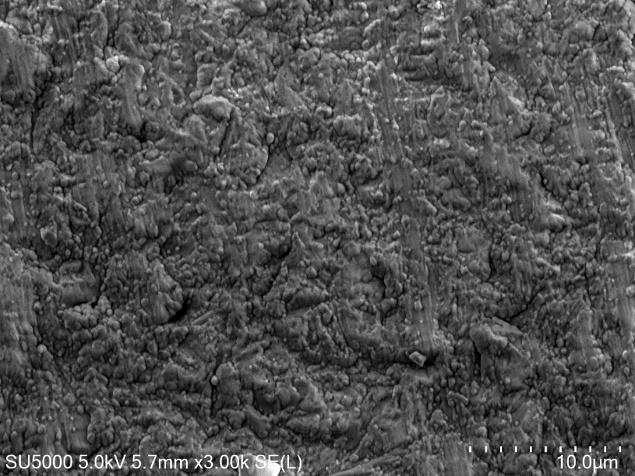


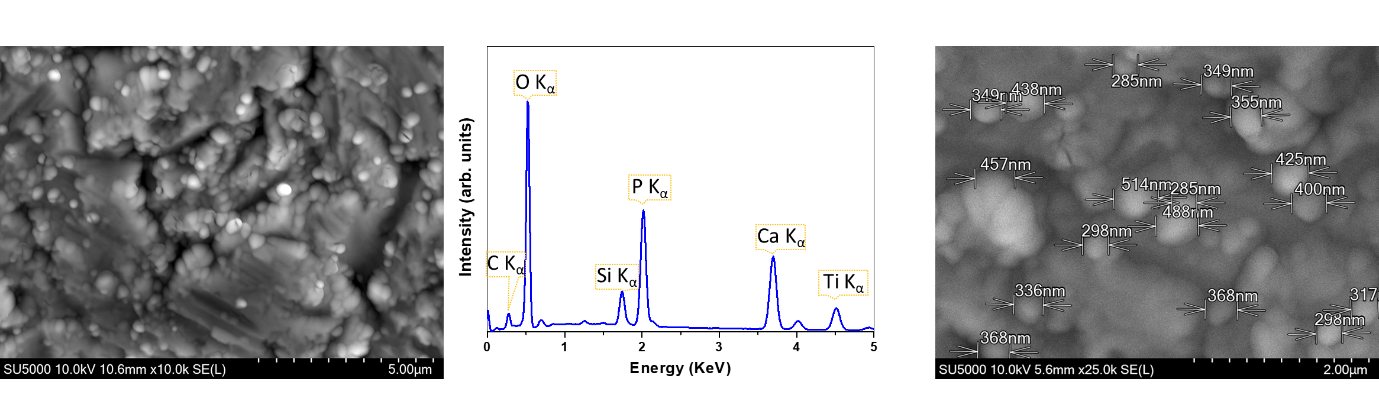


**(e)**

**(d)**

**(c)**

**Supplementary Information Fig. 1** SEM images taken at day 0 from one random location on (a) TI and (b) HA-coated Ti substrate at 3 k magnification. Scale bar represents 10 µm distance within image. SEM/SEM-EDX images taken at day 0 from one random location on HA-coated Ti substrate (c) at 10 k magnification (scale bar represents 5 µm distance within image), (d) corresponding EDX spectra and (e) various HA particle sizes at 25 k magnification (scale bar represents 2 µm distance within image)

**Supplementary Information Fig.** **2** Stack of averaged processed Raman spectra (n = 9) taken at day 0 prior to cell-seeding from three random locations on; (a) TI, (b) TI-HA-AD and (c) TI-HA-ANN. Grey solid line shows Raman peak at ~960 cm^-1^ at day 0 for TI-HA-AD and TI-HA-ANN

**Supplementary Information Fig. 3** Stack of averaged processed Raman spectra (n = 9) taken after exposure to medium under standard conditions for 28 days from three random locations on; (a) TI, (b) TI-HA-AD and (c) TI-HA-ANN. Grey solid line shows Raman peak at ~960 cm^-1^ at day 28 for TI-HA-AD and TI-HA-ANN

**Supplementary Information Fig. 4** Stack of averaged processed Confocal-Raman spectra (n = 36) from the nuclei of three random single U-2 OS cells on quartz substrates. Cells derived from TI over 28 days in culture under standard conditions; (a) day 7, (b) day 14, (c) day 21 and (d) day 28

**
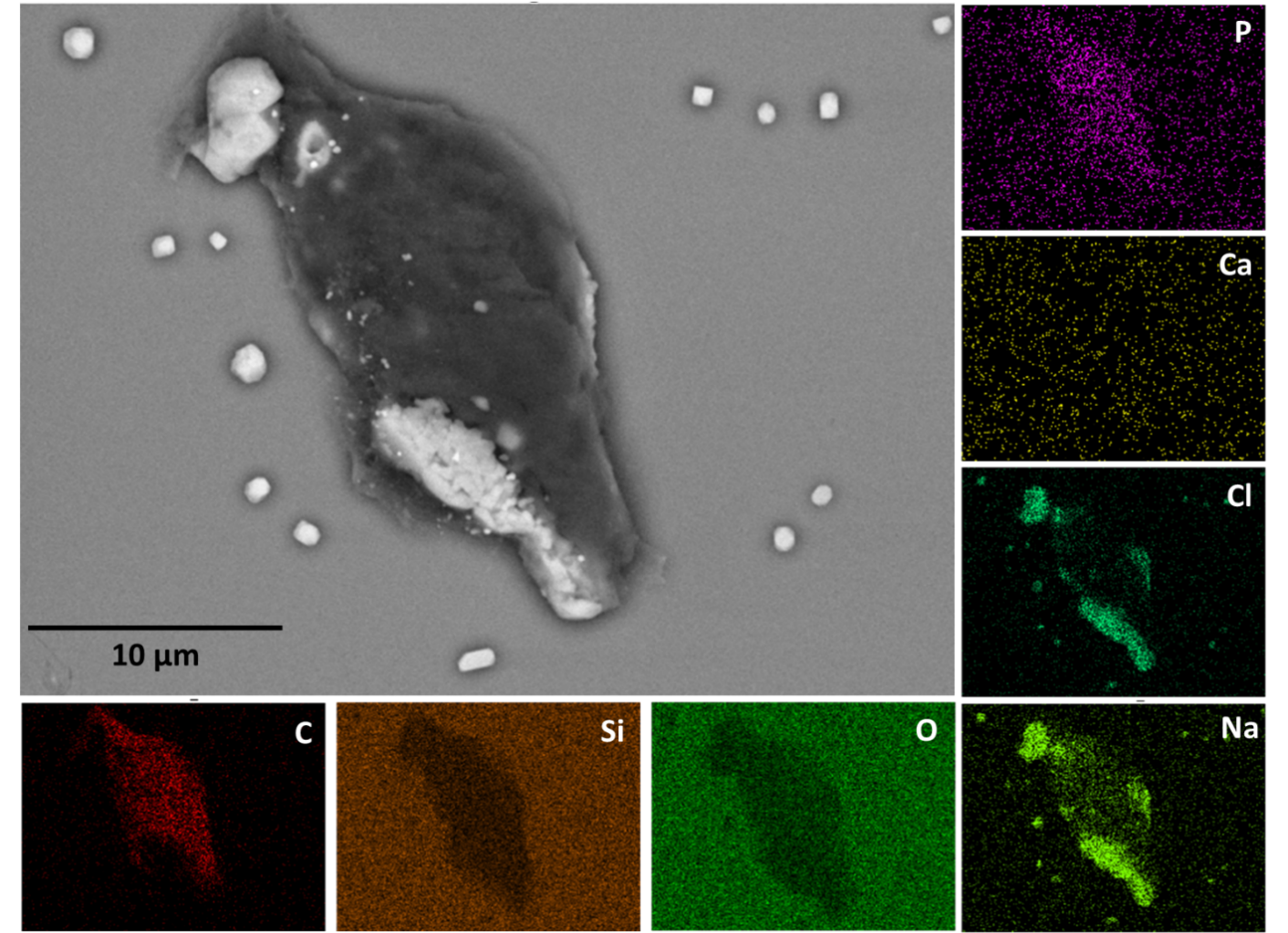
**

**Supplementary Information Fig. 5** SEM image (main image) along with corresponding elemental maps (P, Ca, Cl, Na, C, Si, O and Na) from quartz substrate for single U-2 OS cell derived from TI after 28 days in culture under standard conditions. Scale bar represents 10 µm distance within main image. Dots shown in Ca map are attributable to noise only

**
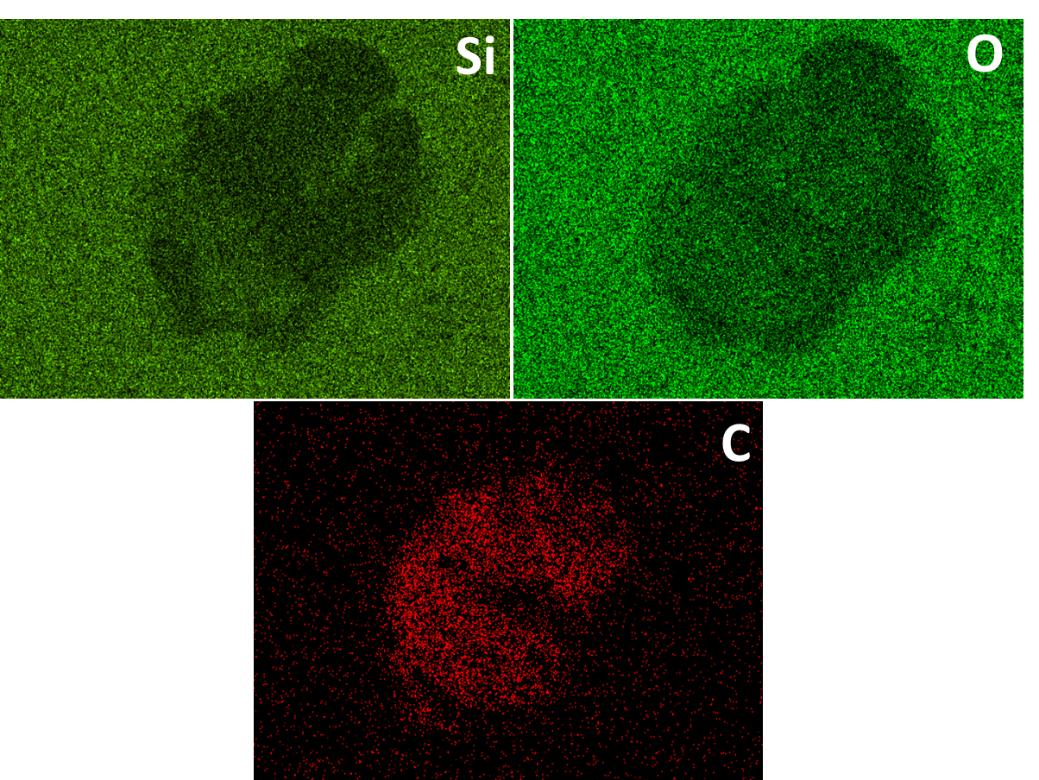
**

**Supplementary Information Fig. 6** Additional elemental maps for Si, O and C from quartz substrate for single U-2 OS cell derived from TI-HA-AD after 28 days in culture under standard conditions, as shown in SEM image in Fig. 7-8 (main manuscript)

**
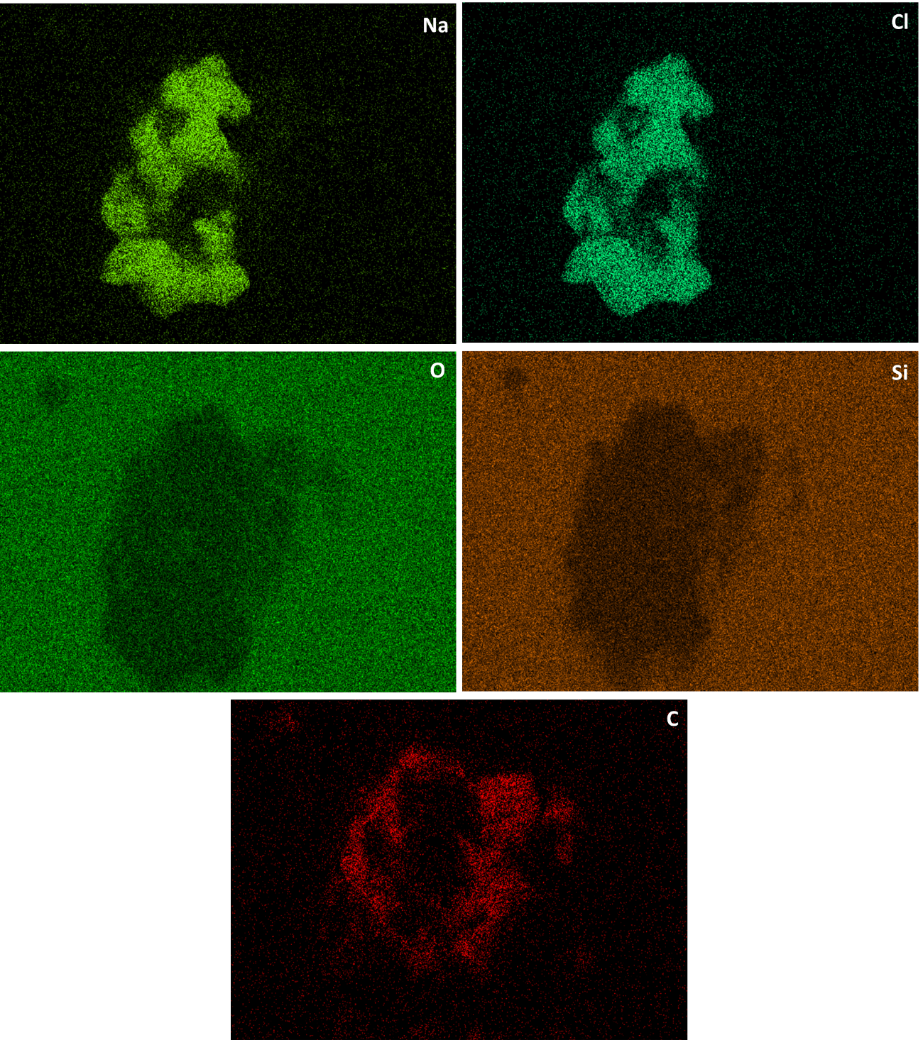
**

**Supplementary Information Fig. 7** Additional elemental maps for Na, Cl, O, Si and C from quartz substrate for single U-2 OS cell derived from TI-HA-ANN after 21 days in culture under standard conditions, as shown in SEM image in Fig. 9 (main manuscript)

**
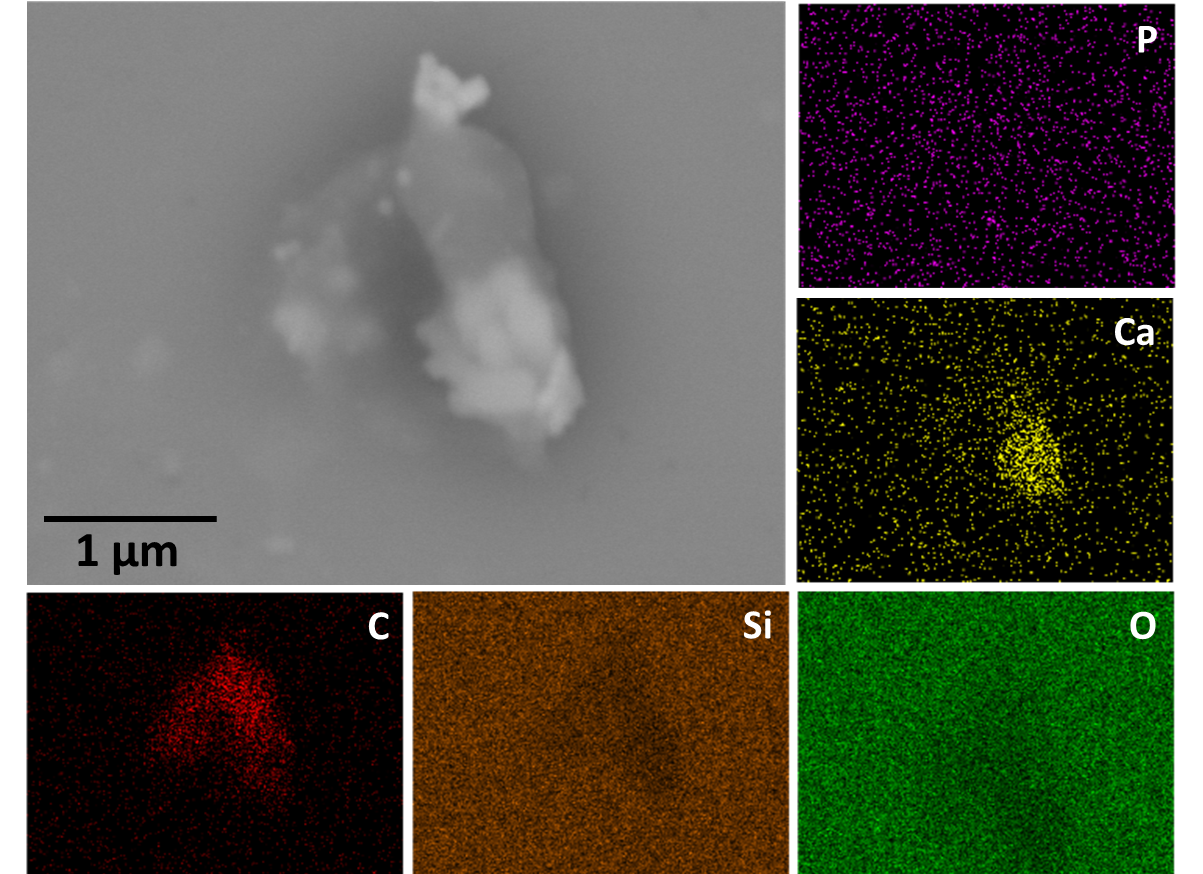
**

**Supplementary Information Fig. 8** SEM image (main image) along with corresponding elemental maps (P, Ca, O, Si and C) from quartz substrate for single U-2 OS cell derived from TI-HA-ANN after 28 days in culture under standard conditions. Scale bar represents 1 µm distance within main image

**
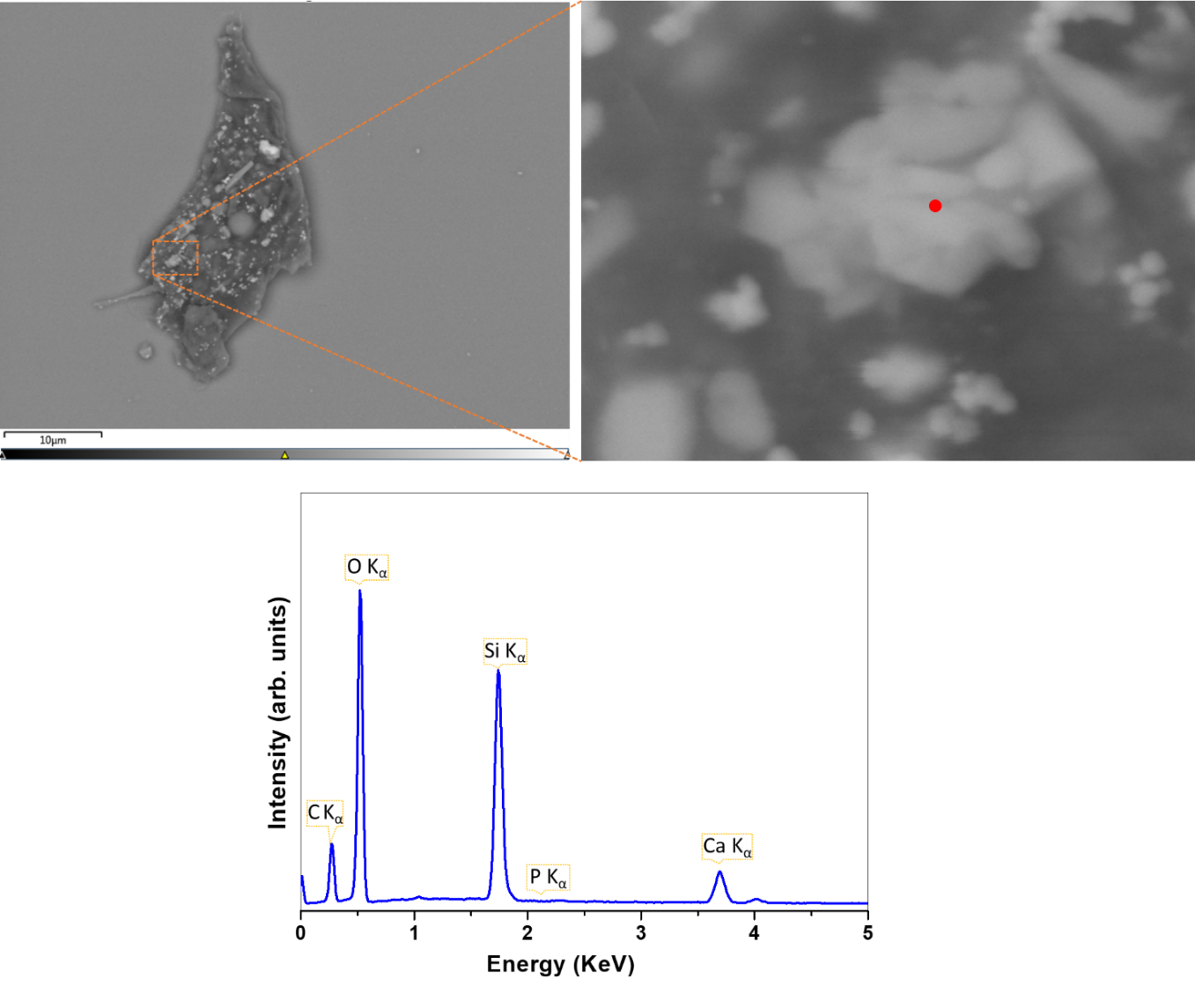
**

**(c)**

**(b)**

**(a)**

**Supplementary Information Fig. 9** (a) SEM image (main image) along with additional SEM-EDX information from quartz substrate for single U-2 OS cell derived from TI-HA-ANN after 28 days in culture under standard conditions, (b) zoomed-in SEM image from (a) and (c) point spectra corresponding to red dot within (b). Scale bar represents 10 µm distance within main image

**
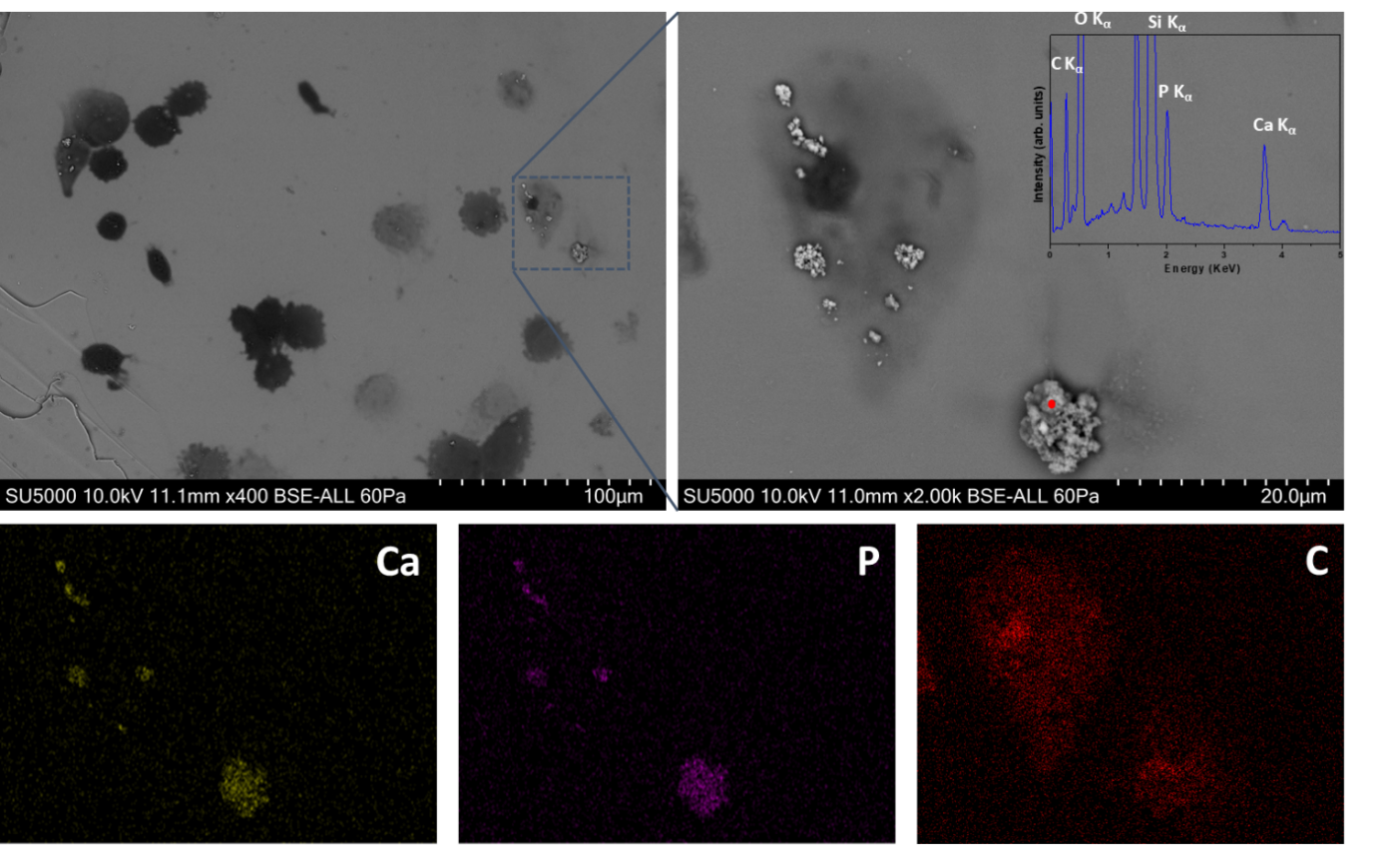
**

**(b)**

**(a)**

**Supplementary Information Fig. 10** (a) SEM image (main image) with scale bar 100 µm distance within image along with corresponding elemental maps (Ca, P and C) from quartz substrate for single U-2 OS cell derived from TI-HA-ANN after 28 days in culture under standard conditions, (b) zoomed-in SEM image from (a) with scale bar 20 µm distance within image along with point spectra corresponding to red dot within image

__________________________________________ END___________________________________________
